# A Knowledge-Driven Approach for 3D High Temporal-Spatial Measurement of an Arbitrary Contouring Error of CNC Machine Tools Using Monocular Vision

**DOI:** 10.3390/s19030744

**Published:** 2019-02-12

**Authors:** Xiao Li, Wei Liu, Yi Pan, Jianwei Ma, Fuji Wang

**Affiliations:** Key Laboratory for Precision and Non-traditional Machining Technology of the Ministry of Education, Dalian University of Technology, Dalian 116024, China; melixiao@mail.dlut.edu.cn (X.L.); 418668164@mail.dlut.edu.cn (Y.P.); mjw2011@dlut.edu.cn (J.M.); wfjsll@dlut.edu.cn (F.W.)

**Keywords:** contouring performance, machine tool accuracy, monocular vision, image processing, contouring error

## Abstract

Periodic health checks of contouring errors under unloaded conditions are critical for machine performance evaluation and value-added manufacturing. Aiming at breaking the dimension, range and speed measurement limitations of the existing devices, a cost-effective knowledge-driven approach for detecting error motions of arbitrary paths using a single camera is proposed. In combination with the PNP algorithm, the three-dimensional (3D) evaluation of large-scale contouring error in relatively high feed rate conditions can be deduced from a priori geometrical knowledge. The innovations of this paper focus on improving the accuracy, efficiency and ability of the vision measurement. Firstly, a camera calibration method considering distortion partition of the depth-of-field (DOF) is presented to give an accurate description of the distortion behavior in the entire photography domain. Then, to maximize the utilization of the decimal involved in the feature encoding, new high-efficient encoding markers are designed on a cooperative target to characterize motion information of the machine. Accordingly, in the image processing, markers are automatically identified and located by the proposed decoding method based on finding the optimal start bit. Finally, with the selected imaging parameters and the precalibrated position of each marker, the 3D measurement of large-scale contouring error under relatively high dynamic conditions can be realized by comparing the curve that is measured by PNP algorithm with the nominal one. Both detection and verification experiments are conducted for two types of paths (i.e., planar and spatial trajectory), and experimental results validate the measurement accuracy and advantages of the proposed method.

## 1. Introduction

Nowadays, as high-end equipment places ever-increasing demands on the accuracy of parts, the manufacturing quality of components is directly related to the performance (i.e., static and dynamic performance) of the machines utilized on the production line. To meet these requirements, measurement is inseparable from any basic or complex CNC machine tool machining process. The conventional post-inspection method is too late for the needs of value-added manufacturing, which drives us to evaluate machine performance before machining to determine or improve the processing capacity. Given the fact that workpieces are dynamically machined, the contouring performance, the capacity to accurately run a given trajectory, is a foundation for the evaluation of the dynamic behavior, especially in high-speed conditions. Thus, the periodic health check of contouring performance in unloaded conditions is critical for maintaining and improving parts’ accuracy [1].

Recently, the International Organization for Standardization (ISO) has collected a variety of machine tool performance testing equipment and specifications for different types of machine tools, formulating a series of norms. For instance, ISO 230 [2] (parts 1–11) specifies the test code for vertical-spindled CNC machines, while the equivalent standard ISO 13041 [3] (parts 1–8) and ISO 10360 [4] (parts 1–6) consider the testing of horizontal spindled machines and coordinate measuring machines (CMMs), respectively. In terms of the ISO 230 series, this year, the ISO/TR 230-11:2018 [5] norm was released to specify the measurement devices suitable for geometry testing of machine tools. To summarize the state-of-the-art ISO standards and research studies, equipment used for dynamic performance assessment under unloaded conditions can be categorized into telescoping ballbar [6,7], cross-grid encoder [8,9], R-test [10,11], laser tracker [12,13] and machine vision technology [14,15,16].

The telescoping ballbar was invented by Jim Bryan [17,18] at the Lawrence Livermore Laboratory (Livermore, CA, USA). It enables health checks of both the static and dynamic properties of a machine by circular interpolation. The corresponding calibration procedures have been included in the newly revised version of ISO 10791-6:2014 [19]; besides, ballbar test results are processed in accordance with ISO 230-4 [20] and ASME B5.54 [21]. Many companies have already commercialized this device, for instance, both Renishaw plc (London, UK) and Heidenhain Ltd. (Berlin, Germany) sell their own brands of DBB instruments and software. Meanwhile, other variants of this kind of measurement equipment have also appeared (i.e., laser ballbars (Optodyne, Inc., Rancho Dominguez, CA, USA)). With the application of telescoping ballbars, a compensation file is generated to improve dynamic performance of machine tools by optimizing the servo controllers. Recent research works based on ballbar devices were mainly dedicated to the modelling and identification of the two types of geometric errors in rotary axes: the position-dependent geometric errors (PDGEs) [22] and the position-independent geometric errors (PIGEs) [16,23]. This quick and efficient diagnostic device is easy to use, however, due to the mechanical structure and the one-dimensional measurement property, the measurable tool path is limited to a circle with a discretely adjustable radius ranging from 50–1000 mm. Furthermore, small ballbar radii that can well highlight the dynamic performance (e.g., servo mismatch) of the machine is not available.

Weikert [24] and Bringmann [25] proposed the ‘R-Test’. Compared with the telescoping ballbar which measures the sphere displacement in one-direction (i.e., the bar direction), the R-test device measures the three-dimensional center location of the master ball by several contact or noncontact [26] displacement sensors. R-test devices are now commercialized by IBS Precision Engineering (Eindhoven, The Netherlands) and Fidia (Turin, Italy). Currently, ISO TC39/SC2/WG3 has discussed the inclusion of the R-test in the revision of ISO 10791-6 [19]. Scholars have made numerous investigations on this method. In [27], a similar diagnostic instrument composed of capacitance sensors, the so-called ‘Capball’, is proposed. Based on the R-test measurement, Zhong [11] presented a measurement method for evaluating the dynamic characteristics of a five-axis machine. The results indicate that the S trajectory test can well reflect the dynamic performance. Christian [10] proposed a method for measuring the thermo-elastic deviation of a five-axis machine. Based on an R-test device, the geometric errors are identified by the proposed mathematical model. The measurement is applied repeatedly over three days to analyze the correlation of the measured errors and the temperature. This metrology provides an efficient way for calibrating rotary and linear axes of three-, four-, or five-axis machining centers. However, similar to the telescoping ballbar, the acceptable tested trajectory is limited to a circular space. What’s more, this circular trajectory is interpolated by the linear axes synchronizing with the rotary axes, hence, error motions of irrelevant axis would be introduced.

Another contact-free optical equipment, the cross-grid encoder, was proposed by Heidenhain Ltd. It enables the contouring performance detection of a two-dimensional (2D) free-form trajectory using diffraction-based technology, and results reported by this device conform to the ISO 230-4 [20] and ISO 10791-6 [19] norms. More recently, research works on this equipment are mainly focused on the detection of both quasi-static [8] and dynamic errors [28]. This diagnostic strategy breaks the measurable trajectory limitations of the abovementioned telescoping ballbar test, however, in practical implementation, a precise scanning gap of 0.5 ± 0.05 mm and fixed angle of 24° are required to obtain finer measuring signals, resulting in its inability to evaluate the contouring accuracy of a rotary axis. Besides, extreme care must be taken during measurement to avoid damaging the grating system.

Laser trackers, which were patented in 1986 [29], allow the 3D positioning of a moving target with an accuracy of a few micrometers. This type of device has been commercialized by Leica Geosystems (Unterentfelden, Switzerland; ± 15 μm + 6 μm/m for the Leica AT 901-B), API (Rockville, MD, USA) and Faro (Exton, PA, USA). Additionally, laser tracers [30] are designed for measuring error motions in rotational axes. Over the past decades, much of the research work in this field has focused on its applications in large-scale dimensional metrology (e.g., aircraft wings and ship hulls), as well as the error mapping of both CMMs and machine tools [31]. However, due to the fact its measurement errors are mainly due to the imperfections of the rotational axes, hence, the 3D values measured by a single laser tracker [32] are insufficient to calibrate high accuracy machine tools with small geometric errors. To address the problem, the new developed multi-laser trackers-based measurement, and the multi-lateration-based scheme [30] have become the mainstreams of current research. At present, these costly and line-of-sight-dependent instruments are mainly used for checking the static and quasi-static performance of machine tools, and their acceptance by end-users to evaluate the dynamic contouring errors is thus difficult.

In summary, for the aforementioned existing single devices, the problem of high accuracy 3D assessments of the contouring errors of an arbitrary trajectory remains unanswered. To solve this problem, a binocular vision-based scheme was proposed in our previous work [15], which realizes spatial contouring error detection of a small-scale equiangular spiral with a range of 45 mm × 45 mm at a maximum feed rate of 2 m/min. Compared with the existing vision-based work [14], satisfactory 3D contouring error detection is achieved. However, the use of two cameras increases the measurement costs, as well as the difficulty in measuring target changes which involve a change in distance under condition that the common DOF is small. As a result, only a 2D trajectory is used for experiments. Besides, in comparison with the state-of-the-art non-vision metrologies, the working range and measurable speed are not high enough. Thus, in [33], we extended the working range and measuring speed of a telecentric optical system, but with this system only 2D contouring errors can be detected. For a single camera equipped with a zoom lens, it has the advantages over binocular vision methods of a wide field of view (FOV), high DOF and multi-dimensional measurement. Thus, to further extend the capability of the vision system, a cost-effective monocular vision-based 3D method is proposed. With the proposed method, the 3D contouring performance of a large-sale arbitrary path in relatively high-dynamic conditions can be evaluated by using PNP algorithm. For metrology, uncertainty analysis is necessary to ensure the measurement accuracy [34]. Especially for the vision measurement method which involves many links. Currently, research in this field falls into two categories: theoretical works [35] and experimental works [36]. The former meet the vision measurement requirements by selecting the optimal parameters through theoretical uncertainty analysis (e.g., mathematical derivation and simulation analysis [37]). For the other direction, the vision measurement accuracy is enhanced by handling the major factors that affect vision measurement uncertainty (e.g., imaging configuration [33], camera calibration [38] and image processing [39]), and returned results are compared with known high accuracy values. In light of this, with the aim of improving the vision measurement accuracy, innovative works focus on the camera calibration improvement, the accurate and efficient image processing, and measurement capability enhancement of single vision measurement.

The rest of paper is organized as follows: in Section 2, to improve the vision measurement accuracy, a calibration method considering distortion partition of the DOF is presented to correct image distortion in the entire photography domain. Section 3 details the high-accuracy image acquisition of the machine tool movement, as well as the image processing for automatic distinguishing and locating the high-efficient encoding markers. Section 4 introduces the knowledge-driven approach for the 3D high temporal-spatial measurement of an arbitrary contouring error using PNP algorithm. In Section 5, contouring error detection tests of two types of paths are preformed to validate the advantages of the proposed method, also the effectiveness and accuracy of the proposed vision method are verified. Finally, in Section 6, conclusions are summarized.

## 2. Principle for Contouring Error Detection Using a Single Camera

### 2.1. Measurement System and Principle

**Figure 1:**
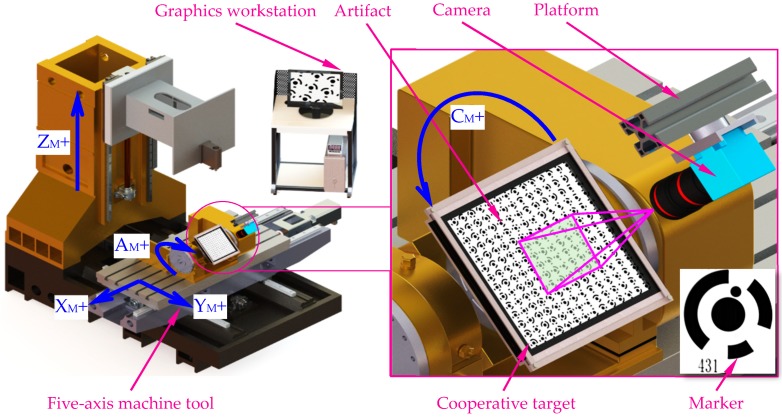
Schematic diagram of the measurement system.

The monocular vision-based contouring error evaluation system (Figure 1) is composed of an industrial camera equipped with an ordinary lens for image acquisition, a platform for adjusting camera pose, a large-size cooperative target that characterizes machine motion, and a graphic workstation for developing image processing and contouring error detection algorithms. One difference from our previous study [15] is that only a single low resolution camera is enough to precisely measure contouring errors in relatively high feed conditions. Another difference is the newly cooperative target designed as a priori knowledge for large-scale contouring performance evaluation, in which high-efficient encoding rule is adopted to distinguish 1024 features.

In this paper, the error motions of a path can be calculated by processing and analyzing the position of features in image sequence. The objective of this paper is to achieve the 3D measurement of an arbitrary large-scale contouring error in relatively high-dynamic conditions using a single camera. Therefore, aiming at improving the accuracy, efficiency, and measurement capability of the proposed method, we perform the following tasks: firstly, combined with the line control field, a camera calibration method considering the distortion partition of the DOF is presented to improve the pixel positioning accuracy of the features (Section 2.2); thereafter, the CNC machine is triggered manually after the workstation allocates memory for image acquisition, and the image sequence of movement cooperative target is acquired by the camera (Section 3.1). After that, in image processing, with the decoding method based on finding the best starting bit proposed in this paper, features in the image sequence can be efficiently distinguished and located (Section 3.2). Subsequently, after selecting the optimal PNP algorithm (Section 4.1) by accuracy comparison analysis, the time-varying point pairs in the object and the image spaces with known position are used to solve the spatial trajectory. Finally, the contouring error can be obtained by comparing the difference between the detected path and the nominal one (Section 4.2).

### 2.2. Camera Calibration Method Considering the Distortion Partition of DOF

**Figure 2:**
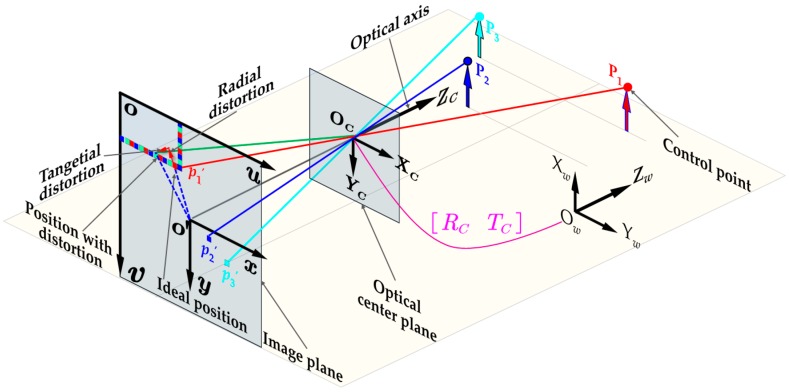
Imaging geometry for central perspective projection.

As shown in Figure 2, $O_{C}X_{C}Y_{C}Z_{C}$ is the camera coordinate system (CCS) that use the optic axis as the Z axis. The 2D pixel coordinate system (PCS) ouv whose *u*- and *v*-axis are parallel to that of the $O_{C}X_{C}Y_{C}Z_{C}$ is located in the upper left element of the image. A space point $P(\begin{array}{ccc} X_{w} & Y_{w} & Z_{w} \end{array})$ in world coordinate system (WCS) is projected on an image point $p(\begin{array}{cc} u_{n} & v_{n} \end{array})$ through the optical center (camera aperture) $O_{C}$, the camera model can be given by [40]:

(1) $$Z_{c}\left[\begin{array}{c} u_{d}\\ v_{d}\\ 1 \end{array}\right]=\underset{K}{\underset{︸}{\left[\begin{array}{ccc} f_{x} & 0 & u_{0}\\ 0 & f_{y} & v_{0}\\ 0 & 0 & 1 \end{array}\right]}}\cdot \underset{M}{\underset{︸}{\left[\begin{array}{cc} R_{C} & T_{C}\\ 0^{T} & 1 \end{array}\right]}}\cdot \left[\begin{array}{c} X_{w}\\ Y_{w}\\ Z_{w}\\ 1 \end{array}\right]$$

where $p^{\prime }(\begin{array}{cc} u_{d} & v_{d} \end{array})$ depicts the pixel coordinates with distortion; $\left(\begin{array}{cc} f_{x} & f_{y} \end{array}\right)$ represent the equivalent focal length in x and y directions, respectively. $\left(\begin{array}{cc} u_{0} & v_{0} \end{array}\right)$ describe the image center. The above-mentioned parameters together form the intrinsic matrix K. While the extrinsic matrix M which consists of $R_{C}$ and $T_{C}$ representing the transformation matrix between the WCS and CCS $O_{C}X_{C}Y_{C}Z_{C}$. In this paper, the 3D measurement capability of a single camera is guaranteed by the Perspective-n-Point (PNP) algorithm. This feature points-based pose estimation problem was proposed by Fishchler [41]. As shown in Figure 2, the PnP problem aims to calculate the six pose parameters (i.e., rotation $R_{C}$ and translation $T_{C}$ matrixes) of a calibrated camera in the WCS from *n* (n>3) known 3D points $P_{i}$ and their 2D projections $p_{i}{}^{\prime }$ in the image.

However, because of the complexity of the optical system, the imperfect imaging of the lens may lead to mapping errors $\left(\begin{array}{cc} \delta_{x} & \delta_{y} \end{array}\right)$. Thus, the first, and also the most important step, is to calibrate the parameters in the imaging model, since for close-range photogrammetry, the nonlinear imaging lens distortion, i.e., radial distortion and tangential distortion, is the main contributor to the measurement uncertainty. Therefore, to meet the high precision measurement requirement, the precise correction of these two manufacturing and assembly induced errors is mandatory. According to Brown’s model [42], imaging distortion for traditional lenses (i.e., ordinary zoom or prime lens) satisfies:

(2) $$\{\begin{array}{c} u_{d}=u_{n}+\delta_{x}\\ v_{d}=v_{n}+\delta_{y}\\ \delta_{x}=u_{n}\cdot (k_{1}+k_{2}\cdot r^{2}+k_{3}\cdot r^{4}+\cdots )+\left[p_{1}\cdot (r^{2}+2\cdot u_{n}^{2})+2p_{2}\cdot u_{n}\cdot v_{n}\right]\cdots \\ \delta_{y}=v_{n}\cdot (k_{1}+k_{2}\cdot r^{2}+k_{3}\cdot r^{4}+\cdots )+\left[p_{2}\cdot (r^{2}+2\cdot u_{n}^{2})+2p_{1}\cdot u_{n}\cdot v_{n}\right]\cdots \end{array}$$

where $p(\begin{array}{cc} u_{n} & v_{n} \end{array})$ depict the pixel coordinates without distortion. $k_{1}$, $k_{2}$ and $k_{3}$ describe the radial distortion coefficients, respectively; the $p_{1}$ and $p_{2}$ represent the first- and second-order tangential distortion coefficients. However, for traditional camera calibration methods, distortion coefficients are calculated with K and M by minimizing the reprojection error, but these parameters will be coupled with each other. Consequently, a small reprojection error may occur when the distortion parameters are not well estimated. Therefore, to improve the camera calibration accuracy, distortion coefficients should be calculated separately. Furthermore, the existing calibration methods mainly focus on the use of a set of distortion coefficients to represent the distortion behavior of the imaging domain [40], or the solution of the distortion of different depth object planes [42,43], when in fact, distortion in the DOF is position-dependent. Hence, for high accuracy distortion correction, the distortion behavior in the DOF needs to be processed more finely. For this intention, a camera calibration method considering the distortion partition of the DOF is proposed, of which the basic idea is to extend the equal-radius partitioned 2D distortion to the 3D imaging domain, and then to separately calibrate the distortion using plumb-line method [42], after locking the obtained optimal distortion parameters in each partition, extrinsic parameters can be optimized.

**Figure 3:**
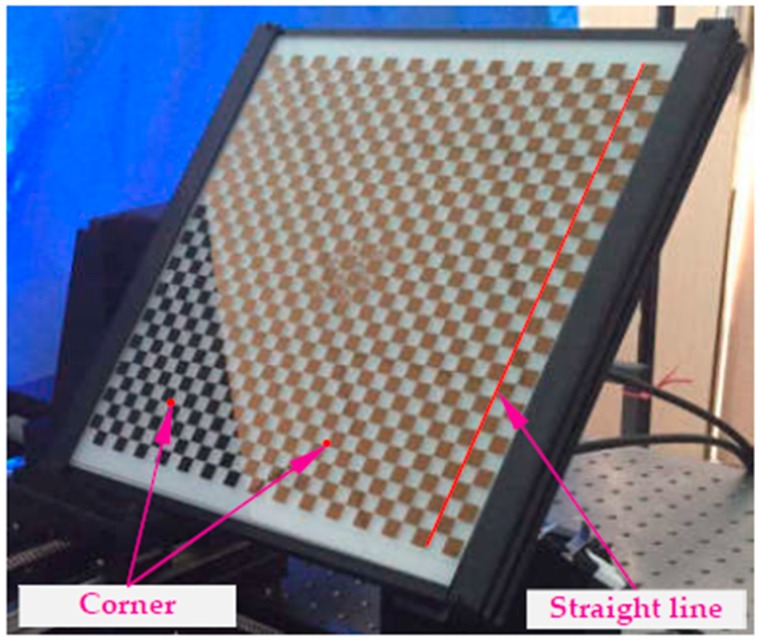
Control field for distortion correction and camera calibration.

To remove the coupling effect of multiple parameters, the line control field is designed and then applied to calibrate the image distortion separately using the plumb-line method. As shown in Figure 3, the corner of the control field is used to calibrate the camera intrinsic parameters [40] to calculate and adjust the pose of the target using the PNP algorithm, while straight lines are utilized to separately calibrate the image distortion of the image. Good pattern manufacturing quality is the premise for high-accuracy camera calibration. To this end, the lithography process is used to make patterns, the linewidth resolution accuracy can be guaranteed to be less than 1.0 μm. Besides, the distance error between two corners is less than 1.5 μm. In terms of partition, firstly, for a single 2D object plane perpendicular to the optical axis, the equal-radius partition method is presented to calculate the distortion in a more accurate way. Considering that the circularly and symmetrically distributed distortion varies with the increase of the distortion radius, the minimum distortion tolerance for image central area is taken as the threshold to calculate the partition radius $r_{p}$, and the number of subregions n can be solved by: $n\cdot r_{p}\leq r_{\max}\leq (n+1)\cdot r_{p}$, where $r_{\max}=\sqrt{{\left(I_{w}-u_{0}\right)}^{2}+{\left(I_{l}-v_{0}\right)}^{2}}$ is the maximum distortion radius; $I_{w}$ and $I_{l}$ depict the image width and image length, respectively.

According to [44], when the lens is focused at an unknown depth of s, the radial $k_{i}^{s,s_{n}}$, and tangential $p_{j}^{s,s_{n}}$ distortion parameters at an arbitrary depth $s_{n}$ can be computed by the calibrated distortion parameters of two known different depths $s_{m}$ and $s_{k}$, which can be given by:

(3) $$\left\{ \begin{array}{ccc} k_{i}^{s,s_{n}}=\alpha_{s_{n}}\cdot \frac{c_{s_{n}}^{2}}{c_{s_{m}}^{2}}\cdot k_{i}^{s,s_{m}}+(1-\alpha_{s_{n}})\cdot \frac{c_{s_{n}}^{2}}{c_{s_{k}}^{2}}\cdot k_{i}^{s,s_{k}} & & i=1,2,3.\\ p_{j}^{s,s_{n}}=p_{j}^{s,s_{m}}=p_{j}^{s,s_{k}} & & j=1,2.\\ \frac{1}{s_{n}}+\frac{1}{c_{s_{n}}}=\frac{1}{f} & & \end{array}\right.$$

where $\alpha_{s_{n}}=\frac{s_{k}-s_{n}}{s_{k}-s_{m}}\cdot \frac{s_{m}-c}{s_{n}-c}$, $s_{n}$, $s_{m}$ and $s_{k}$ denote the depth of three object planes perpendicular to the optical axis; c, $c_{s_{n}}$, $c_{s_{m}}$ and $c_{s_{2}}$ are the principal distances when the focus plane is at infinity, depth $s_{n}$, depth $s_{m}$ and depth $s_{k}$. $k_{i}^{s,s_{m}}$ and $k_{i}^{s,s_{k}}$ describe ith radial distortion parameters of two known object planes. $p_{j}^{s,s_{m}}$ and $p_{j}^{s,s_{k}}$ describe jth tangential distortion parameters of two known object planes.

According to Equation (1), spatial points satisfy:

(4) $$\{\begin{array}{c} x=f\cdot \frac{X_{m}}{Z_{m}}=f\cdot \frac{X_{k}}{Z_{k}}\\ y=f\cdot \frac{Y_{m}}{Z_{m}}=f\cdot \frac{Y_{k}}{Z_{k}} \end{array}$$

where $\left(\begin{array}{ccc} X_{m} & Y_{m} & Z_{m} \end{array}\right)$ and $\left(\begin{array}{ccc} X_{k} & Y_{k} & Z_{k} \end{array}\right)$ are the coordinates of the two spatial points in the CCS, respectively; accordingly, $\left(\begin{array}{cc} x & y \end{array}\right)$ denote the 2D projection on the image plane expressed in mm. Then, for a partition radius $x^{2}+y^{2}=\rho^{2}$, we have:

(5) $$\{\begin{array}{c} f^{2}\cdot \frac{X_{m}^{2}}{Z_{m}^{2}}+f^{2}\cdot \frac{Y_{m}^{2}}{Z_{m}^{2}}=\rho^{2}\\ f^{2}\cdot \frac{X_{k}^{2}}{Z_{k}^{2}}+f^{2}\cdot \frac{Y_{k}^{2}}{Z_{k}^{2}}=\rho^{2} \end{array}$$

**Figure 4:**
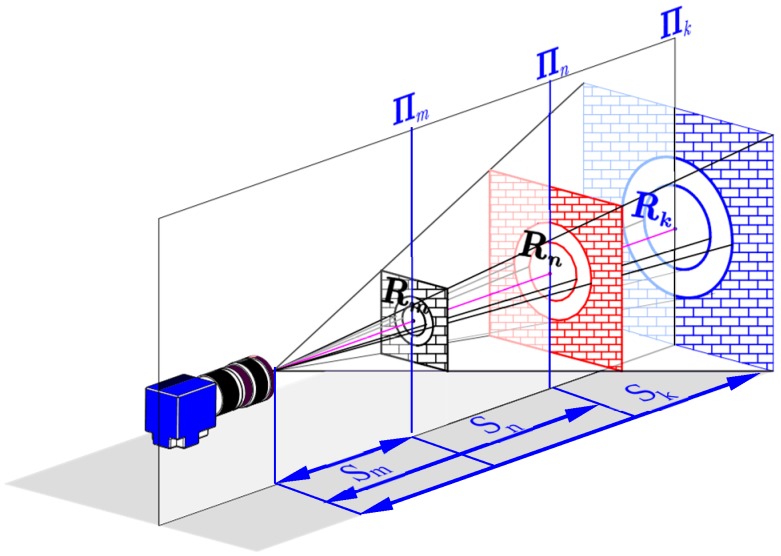
Geometric relationship between the partition radiuses at different object planes.

Solving the equation and re-arranging, we get $f\cdot R_{m}=\rho \cdot Z_{m}$ and $Z_{m}\cdot R_{k}=Z_{k}\cdot R_{m}$. where, $Z_{m}$ and $Z_{k}$ are the depths of the mth ($\Pi_{m}$) and kth ($\Pi_{k}$) object planes in the CCS; $R_{m}$ and $R_{k}$ describe the corresponding partition radius of the two planes; f represents the lens focal length. Let $s_{m}=Z_{m}$ and $s_{k}=Z_{k}$, then the aforementioned in-plane distortion partition is extended to the 3D DOF, as can be shown in Figure 4, based on the gth partition on object plane $\Pi_{m}$ with partition range of $\left[\begin{array}{cc} (g-1)\cdot R_{m} & m\cdot R_{m} \end{array}\right]$, the corresponding partition range on object plane $\Pi_{k}$ and $\Pi_{n}$ at the object distance $s_{k}$ and $s_{n}$ are $\left[\begin{array}{cc} \left(g-1)\cdot (s_{k}\cdot R_{m}/s_{m}\right) & g\cdot (s_{k}\cdot R_{m}/s_{m}) \end{array}\right]$ and $\left[\begin{array}{cc} \left(g-1)\cdot (s_{n}\cdot R_{m}/s_{m}\right) & g\cdot (s_{n}\cdot R_{m}/s_{m}) \end{array}\right]$, respectively. Consequently, the radial kis,sng and decentering pis,sng distortion coefficient under the gth partition of arbitrary object distance $s_{n}$ can be expressed as:

(6) {kis,sng=αsn·csn2csm2·kis,smg+(1−αsn)·csn2csk2·kis,skgi=1,2,3.pjs,sng=pjs,smg=pjs,skgj=1,2.1sn+1csn=1f

The radial (kis,smg and kis,skg) and tangential (pjs,smg and pjs,skg) distortion in each subregion of the two known planes can be estimated by minimizing the straightness error of the corresponding straight lines. Then, based on Equation (6), the distortion partition and corresponding distortion coefficients of arbitrary object distance $s_{n}$ can be determined, which can give an optimal description of the distortion behavior in the DOF. To further improve the calibration accuracy, the determined distortion and the intrinsic parameters of the imaging model are locked, and calibration target (Figure 3) with high-precision grid distance is used to optimize extrinsic parameters:

(7) $$\{\begin{array}{c} E_{depth\_dependent}^{q}(\begin{array}{cc} R_{q} & T_{q} \end{array})=\sum_{g=1}^{m_{g}}distance(H^{-1}(\begin{array}{cccccccc} u_{0} & v_{0} & f_{x} & f_{y} & {}^{g}k_{i} & {}^{g}p_{j} & R_{q} & T_{q} \end{array}))\\ i=1,2,3.\\ j=1,2. \end{array}$$

where, $E_{depth\_dependent}^{q}$ is the cost function of the target at the *q*th pose, $R_{q}$ and $T_{q}$ are the rotation and translation matrix under the *q*th pose to be optimized, kig and pjg represent the radial and tangential distortion parameters of the *g*th distortion partition under the *q*th pose. In this paper, we used Levenberg-Marquardt (LM) algorithm to optimize the objective function. It needs to be emphasized that the constructed distortion calibration model with respect to the entire photography domain is related to the 3D position of a space point. Based on the established camera calibration method, the distortion of an image point can be corrected by selecting the optimal position-dependent distortion model.

## 3. High Precision 3D Positioning of Machine Tool Movement

In this paper, a 3D metrology is proposed to detect contouring errors using a single camera. To enable the working range measurement using a priori geometric information, as well as maintain a high vision measurement accuracy to evaluate the dynamic performance, high-quality image acquisition and high-accuracy image processing should be guaranteed. This section describes these two aspects in detail.

### 3.1. High-Quality Image Acquisition for Machine Tool Movement

**Figure 5:**
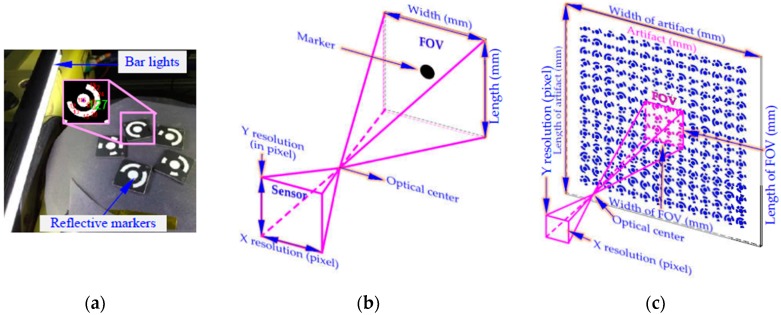
Means for motion description. (**a**) Traditional reflective markers. (**b**) Small-size artifact. (**c**) Large-size artifact with coded markers.

**Figure 6:**
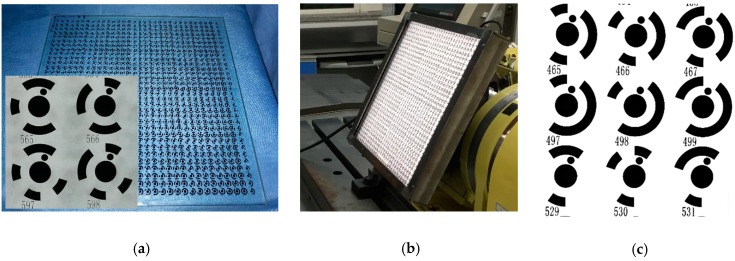
Large-size cooperative target. (**a**) Artifact. (**b**) Cooperative target fixed in the workbench. (**c**) High SNR image of the acquired coded marker.

In practical vision measurement, it is necessary to add features to highlight the information of the object measured to be measured [45]. Thus, to precisely describe the movement information of the machine, markers should be installed at the end of the workbench as an enhanced feature. However, for machine motion capture scheme with bar lights radiating forward light for traditional reflective markers (Figure 5a). The unsatisfactory image quality (e.g., high reflection and image noise) and low marker manufacturing accuracy (shape error larger than 70 μm) limit the high-accuracy contouring error detection. To this end, our previous work [15] proposed a cooperative target based on high-precision lithography and high uniform backward illumination. For such a “what-you-see-what-you-get” measurement scheme (Figure 5b), the wide range measurement capability depends entirely on wide FOV size and large camera resolution. Obviously, this costly measurement scheme is not feasible due to the limitations of the camera hardware. To address the problem, a monocular vision-based 3D high temporal-spatial measurement method for contouring error detection is proposed (Figure 5c). To enhance the marker encoding efficiency, the number of 1024 new fiducial markers (see Section 3.2) is designed and coded on the large-size artifact. As shown in Figure 6, the artifact (Figure 6a) is embedded in a cooperative target (Figure 6b) to describe the motion information. In addition, a flat backlight independent of ambient lighting is employed to enhance the markers to be inspected. A high signal-to-noise-ratio (SNR) value of 38.7 dB can be obtained for the marker image acquired by the proposed method.

**Figure 7:**
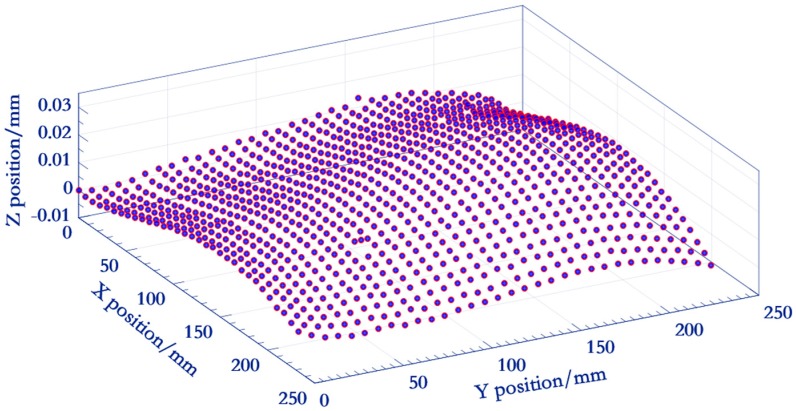
3D geometric relationships between markers.

The photoetching technology (±0.5 μm) ensures the shape error of a single feature less than 1 μm. However, the big warp error of the large-size glass reduces the geometric accuracy among markers [33]. Thus, the 3D position of markers is calibrated by a HEXAGON OPTIV reference instrument (Qingdao, China, measurement error $E_{x}$, $E_{y}$: 0.25 μm + *L*/900, $E_{z}$ = 0.5 μm) as a priori information (Figure 7) to ensure the vision measurement accuracy under a wide range of conditions.

### 3.2. Accurate Encoding and Identification Method for Coded Targets

**Figure 8:**
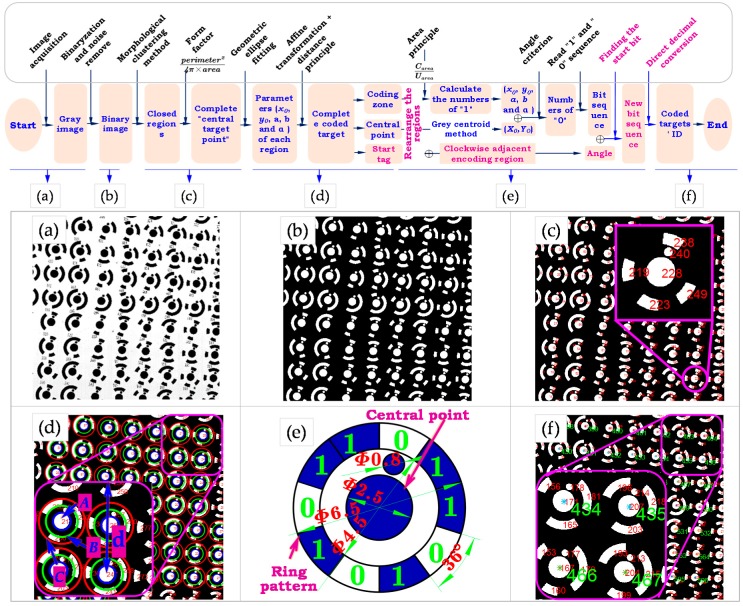
A work flow for the automatic identification and location of coded markers.

As mentioned above, we design a large-size artifact. To make the proposed wide range measurement method feasible, a certain number of fiducial markers that are easy to distinguish from each other should be distributed over a large measurement basis. For this intension, considering the invariant attribute of the radially symmetric form, fiducial marker with ring pattern around the center point is designed as the enhanced feature (Figure 8e). The center point is used to represent the motion information of the worktable; while the ring pattern consists of encoding region (bit sequence “1”) and non-encoding region (bit sequence “0”) serves to distinguish each fiducial marker. To perform large-scale contouring error detection using a priori information, the time-varying fiducial markers need to be accurately and automatically identified. However, for traditional encoding method, the identification number is defined as the smallest decimal value converted from binary sequences after cyclic shift [33,46]. As a result, the utilization of the decimal involved in the encoding process is less than 1/n due to the inefficient encoding rules, where n is the number of bits. Therefore, an efficient encoding and decoding method based on finding the best start bit is proposed. With the method, for each fiducial marker, an auxiliary circular tag is added and pointed to the lowest bit of the binary code. Since the decimal corresponding to the *i*th coded marker is *i*, the effective utilization of the decimal is increased to 100%. Then, the 1024 fiducial markers used in this paper can be encoded by 10 bits, while the traditional method requires at least 15 bits.

After encoding, the coded value can be calculated by the image analysis of the ring pattern defining the code, the main idea of the decoding method is to reorganize the clockwise arranged bit sequence after finding the start bit, and then to identify the coded value by direct decimal conversion. The identification algorithm can be split up into the following steps:

(1) Image preprocessing and geometric ellipse fitting

As shown in Figure 8b, image preprocessing (e.g., binarization, noise rejection) is conducted for the acquired gray image (Figure 8a) first, then the morphological clustering method [47] is combined with the form factor $P_{\min}\leq (perimeter^{2}/4\pi \times area)\leq P_{\max}$ to distinguish the center point and encoding regions. For detailed description, please refer to our previous work [15]. Thereafter, the five parameters $\left(\begin{array}{ccccc} x_{c} & y_{c} & a & b & \theta \end{array}\right)$ of each closed region is obtained by the geometric ellipse fitting method.

(2) Obtain the complete fiducial marker

In practical measurement, imaging parameters of small FOV and low camera resolution are used to improve the measureable traverse speed. Besides, this paper involves the contouring error detection of a real 3D path, hence, the resultant perspective effect increases the difficulty in removing incomplete fiducial marker in the image boundary, as well as in calculating the number of “1” in the encoding region using area criteria. Therefore, the affine transformation is performed to get closed regions perpendicular to the optical axis, which can be given by:

(8) $$\left(\begin{array}{c} x^{\prime }\\ y^{\prime }\\ z^{\prime } \end{array}\right)=\left[\begin{array}{ccc} 1 & 0 & x_{c}\\ 0 & 1 & y_{c}\\ 0 & 0 & 1 \end{array}\right]\cdot \left[\begin{array}{ccc} 1 & 0 & 0\\ 0 & a/b & 0\\ 0 & 0 & 1 \end{array}\right]\cdot \left[\begin{array}{ccc} \cos\theta & \sin\theta & 0\\ -\sin\theta & \cos\theta & 0\\ 0 & 0 & 1 \end{array}\right]\cdot \left[\begin{array}{ccc} 1 & 0 & x_{c}\\ 0 & 1 & -y_{c}\\ 0 & 0 & 1 \end{array}\right]\cdot \left[\begin{array}{c} x\\ y\\ 1 \end{array}\right]$$

where $\left(\begin{array}{ccccc} x_{c} & y_{c} & a & b & \theta \end{array}\right)$ is the five parameters of an ellipse, $\left(\begin{array}{cc} x & y \end{array}\right)$ represents the coordinates of the original image, $\left(\begin{array}{cc} x^{\prime } & y^{\prime } \end{array}\right)$ represents the transformed coordinates corresponding to the original image $\left(\begin{array}{cc} x & y \end{array}\right)$. Then, based on the central positions and radius ratio ($r_{A}:r_{B}:r_{C}=6.5:4.5:2.5$), closed regions attached to the same coded target can be obtained (Figure 8d), and coded targets in the image can thus be distinguished between each other. Thereafter, the complete coded targets are determined by $r_{A}\leq d$, where $r_{A}$ and *d* are determined by the imaging parameters and the physical dimension of the coded target.

(3) Arrange vectors clockwise beginning with the start tag

**Figure 9:**
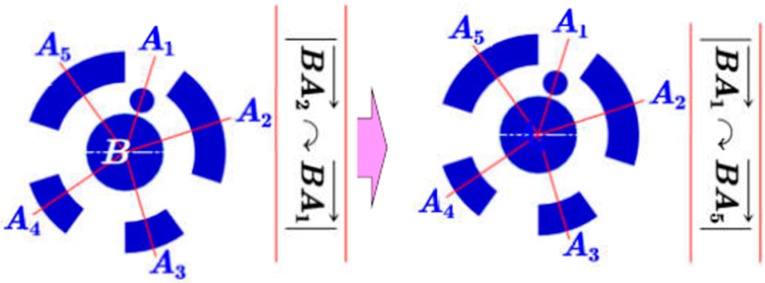
Arranging vectors clockwise beginning with the ‘start tag’.

First, the center of the inner circle, denoted by B, is located by the grey centroid method [48]. For each complete fiducial marker, a set of straight line vectors $\vec{BA_{i}}$ are formed by joining the center point and the centroid of the surrounding encoding regions. Then, straight line vectors are arranged in clockwise $\vec{BA_{2}}\cdots \vec{BA_{5}}\to \vec{BA_{1}}$ (Figure 9). Afterwards, the start tag is found (see Step 1) and considered to be the initial position to rearrange vectors, and we get $\vec{BA_{1}}\cdots \vec{BA_{4}}\to \vec{BA_{5}}$.

(4) Read the binary sequence clockwise

**Figure 10:**
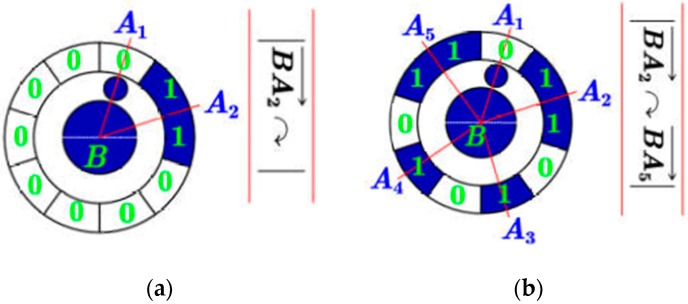
Reading binary sequence clockwise. (**a**) case 1. (**b**) case 2.

It is noted that the ‘start tag’ is not involved in the calculation of “0” and “1”. The numbers of “1” with respect to each encoding region is deduced by the area criterion $C_{area}/U_{area}$, where $C_{area}$ is the area of a encoding region; $U_{area}$ denotes the area of the unit encoding zone which can be described by a binary “1”. Meanwhile, the calculation for the numbers of “0” in the non-encoding regions can be categorized into two cases:

A) Case 1: only one encoding region around the central point
  As shown in Figure 10a, suppose that this encoding region consists of m “1”, then the number of “0” with respect to the non-encoding region is n=10−m, and we get the binary sequence $\underset{m}{\underset{︸}{11\cdots }}\underset{n=10-m}{\underset{︸}{00\cdots }}$.
B) Case 2: more than one encoding region around the central point
  The numbers of “0” in the non-encoding region between two adjacent encoding regions (Figure 10b) can be deduced by $n=C_{angle}/U_{angle}-\left[\left(M_{i}+M_{i+1}\right)/2\right]$. Where $C_{angle}$ describes the angle formed by adjacent two vectors (e.g., $∠A_{2}BA_{3}$), $U_{angle}=10^{{}^{\circ}}$; $M_{i}$ and $M_{i+1}$ are the numbers of “1” in two adjacent encoding regions. By traversing the entire ring pattern, we get the binary sequence of.$\overset{\begin{array}{cc} Read & direction \end{array}}{\underset{\underset{A_{2}}{\underset{︸}{11}}0\underset{A_{3}}{\underset{︸}{1}}0\underset{A_{4}}{\underset{︸}{1}}0\underset{A_{5}}{\underset{︸}{11}}0}{\to }}$.

(5) Calculate the coded value

Through the above calculation, a clockwise binary sequence, denoted by Binary_sequence, starting from the encoding region closest to the start tag is obtained. Then, the decoding method based on finding the start bit is proposed to deduce the identification number.

**Figure 11:**
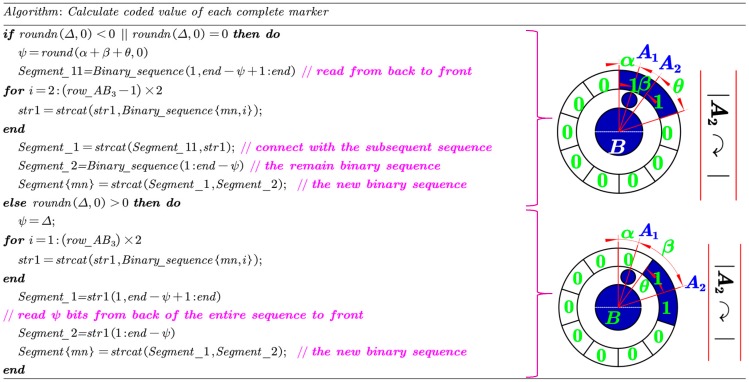
Pseudo-code description of calculating the coded value.

**Figure 12:**
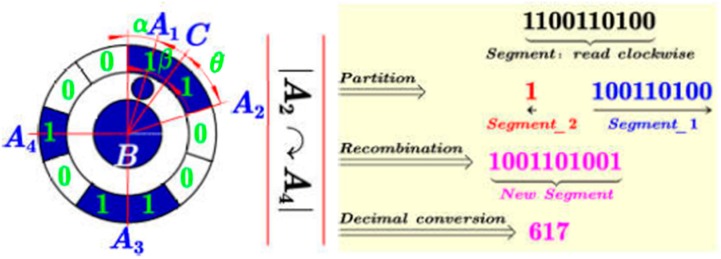
Schematic diagram of coded value calculation when Δ<0 or Δ=0.

**Figure 13:**
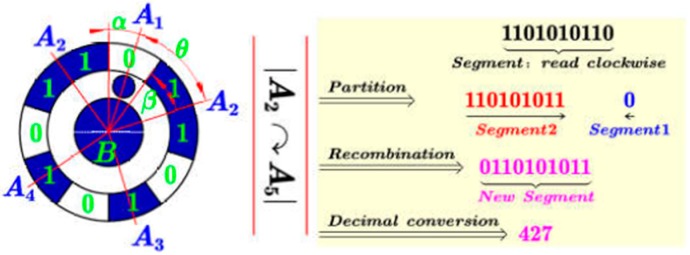
Schematic diagram of coded value calculation when Δ>0.

Let $\Delta =\alpha +\beta /36^{{}^{\circ}}-\theta$, where α=0.5; β describes the angle between the start tag and its nearest encoding region along clockwise direction ($∠A_{1}BA_{2}$ in Figure 11); θ denotes half of the number of “1” contained in the first clockwise encoding region. As illustrated in Figure 11, the coded value can be calculated by the following two cases:

A) Case 1: if Δ<0 or Δ=0
  As shown in Figure 12, Let ψ=roundn(α+β+θ,0), and assume the first encoding region consists of k number of “1”, where k≥ψ. First, ψ numbers of “1” from the back to the front of this sequence are read and then connected with the following sequence to get the Segment_1; while the remain k−ψ number of “1” in the first encoding region is denoted by Segment_2. Finally, the new segment is obtained by connecting Segment_1 to Segment_2.
B) Case 2: Δ>0
  ψ=Δ binary numbers from the back of the entire binary sequence to the front are read to form Segment_1 (Figure 13), and the remain binary sequence is denoted by Segment_2. Then, the new segment is obtained by connecting Segment_1 to Segment_2.

The detailed pseudo-code description of calculating the coded value is given in Figure 11. Finally, the coded target can be decoded by directly converting the new binary sequence to decimal. The Supplementary Materials give an external video for image sequence processing. And all the markers in the image sequence can be accurately identified.

## 4. 3D High Spatial-Temporal Measurement of Large-Scale and Relatively High-Dynamic Contouring Error

### 4.1. Pose Estimation Algorithms Comparison

Currently, PNP algorithms can be classified as closed solution based algorithms (P3P, P4P, P5P), non-iterative algorithms (DLT, TASI) and iterative algorithms. The former two algorithms are easily affected by noise; while the non-iterative algorithm has obvious advantages in measuring accuracy and stability. Research on PNP algorithm mainly focuses on the measurement accuracy, efficiency and stability. To make it work, at least three (or six) control points are required for vision system with known (or unknown) intrinsic parameters. In this paper, three classical algorithms, i.e., ‘DLS’ [49], ‘LHM’ [50] and ‘OPNP’ [51] have been selected and tested to find the most accurate one for practical application.

**Figure 14:**
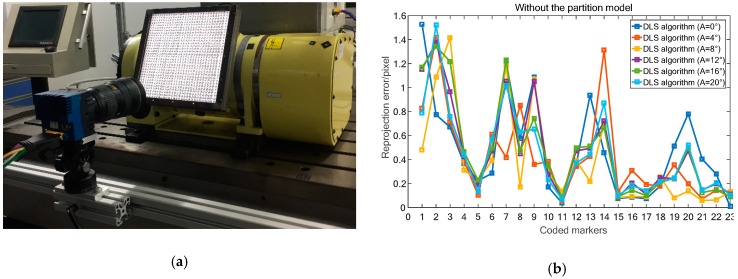

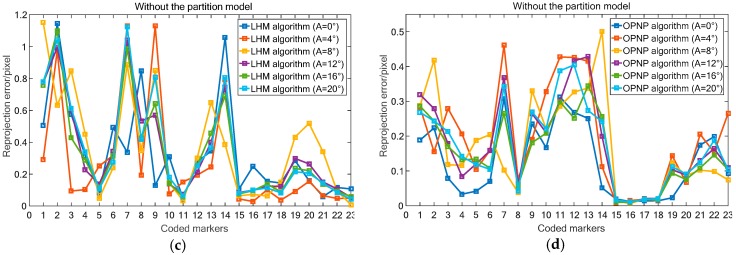
Accuracy comparison of the three algorithms without distortion partition. (**a**) Experimental setup. (**b**) Reprojection error of DLS code. (**c**) Reprojection error of LHM code. (**d**) Reprojection error of OPNP code.

All algorithms were tested by image reprojection errors of the features at a fixed position without considering the proposed distortion partition model. Specifically, the target is driven by the A axis of the machine to rotate to six angular positions (Figure 14a). And, in each stop position, several features are used by the three codes to constructed the pose $\left(\begin{array}{cc} R & T \end{array}\right)$ of a calibrated camera. Thereafter, the whole 23 features are projected back to the image via the $\left(\begin{array}{cc} R & T \end{array}\right)$, and the measurement accuracy of the three algorithms are compared by the reprojection error, which is the image distance between the projected point and a observed one.

As illustrated in Figure 14, the maximum (average) reprojection errors of the six positions using the DLS (Figure 14b) and LHM (Figure 14c) algorithms are 1.52 pixel/0.45 pixel and 1.15 pixel/0.34 pixel. While, the maximum and average projection errors of the OPNP algorithm (Figure 14d) are 0.51 pixel and 0.17 pixel, respectively. Considering the verification results, the OPNP algorithm which returns high-accuracy pose estimation is selected in our paper.

### 4.2. Wide Range Contouring Error Detection

To ensure the vision measurement accuracy high enough to evaluate the contouring error, a high-speed camera with large resolution is needed. However, the FOV, camera resolution and frames per second (FPS) interact with each other [33]:

(9) $$\{\begin{array}{c} FOV/\begin{array}{cc} Camera & resolution=\begin{array}{cc} Spatial & resolution \end{array} \end{array}\\ \begin{array}{cc} Camera & resolution\times FPS(\begin{array}{cc} Time & resolution \end{array})=Bandwidth \end{array} \end{array}$$

where $\begin{array}{cc} Spatial & resolution \end{array}$ is measured in mm/pixel; Bandwidth is defined as the amount of data that has to be transmitted per second. When the camera interface and the capture card are determined, the Bandwidth is a constant. For the “what-you-see-what-you-get” measurement scheme (Figure 5b), the working range entirely depends on the size of the FOV. Hence, to improve the working range while maintaining a satisfactory $\begin{array}{cc} Spatial & resolution \end{array}$ (Equation (9)), the increased camera resolution will reduce the FPS (i.e., working speed/time resolution). Conversely, for constant values of Bandwidth and $\begin{array}{cc} Spatial & resolution \end{array}$, to increase the working speed (i.e., FPS), the camera resolution should be reduced. As a result, the narrow FOV reduces the working range of the vision system. To summarize, the working range and the working speed cannot be simultaneously and greatly improved by simply adjusting the camera imaging parameters. Thus, for further enhancing the measurement ability of the vision system, in combination with a priori geometric constraint (Figure 7), a monocular vision-based 3D high temporal-spatial measurement method is proposed in this paper. The basic idea of the method is to improve the working speed (i.e., FPS) of the vision system by scarifying the FOV and camera resolution, while the wide range measurement capability of the vision device is realized by a priori geometric constraint. Specifically, the FOV and resolution of the camera can be scaled down to only guarantee the proper positioning accuracy of several markers in the image (Figure 5c). As a result, the measurable speed of the vision system can be increased; while, in case of wide range measurement, a measurement fixture with 1024 coded markers on the artifact (Figure 6a) is designed. For the coded markers, one of them is selected as the ‘reference feature’ to represent the whole motion trajectory of the machine, while the others are defined as auxiliary coded markers used for calculating the position of the ‘reference feature’.

Therefore, according to Equation (9), the camera’s frame rate can be increased by scaling down the FOV and camera resolution. Theoretically, image blur can be suppressed by only increasing the frame rate by reducing the camera resolution. But in this paper, to ensure the vision measurement accuracy at low camera resolution, the FOV is also reduced. In practical implementation, a CoaXPress interface camera with full-resolution of 5120 × 5120 pixels is used. The built-in "Region of Interest" (ROI) function enables the camera to send images at faster frame rate by sacrificing the camera resolution:

(10) $$\frac{R_{full}}{F_{full}}=\frac{R_{reduce}}{F_{reduce}}$$

As can be seen from Equation (10), when the full resolution $R_{full}$ is reduced to a low resolution $R_{reduce}$ without changing the focal length of the lens, the camera’s FOV $F_{full}$ will be reduced to $F_{reduce}$ in the same proportion as the camera resolution. Therefore, the spatial resolutions before and after the adjustment of imaging parameters remain unchanged, and the vision measurement accuracy can thus be ensured. Besides, after the adjustment, the FOV can be further reduced by increasing the focal length, and thus the positioning accuracy of the marker in the FOV can be improved due to the enlargement of the spatial resolution of the vision system.

**Figure 15:**
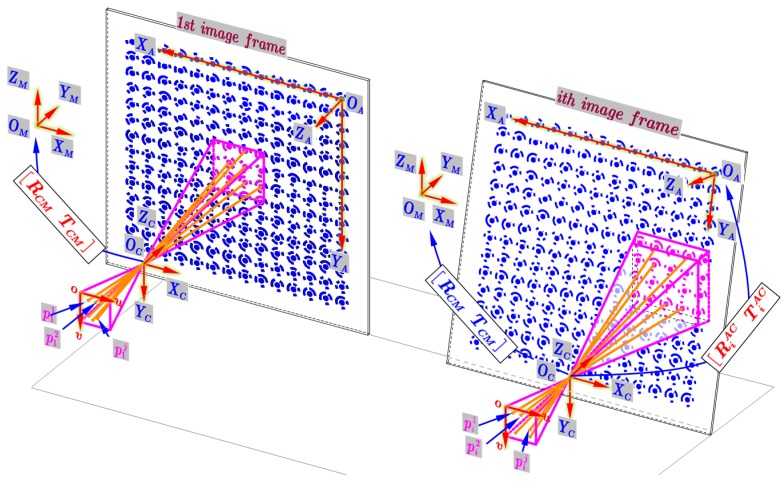
Principle for 3D high temporal-spatial measurement using PNP algorithm.

To begin with, we give definitions of several coordinate systems. As illustrated in Figure 15, during the measurement, the involved coordinate frames consist of artifact coordinate system (ACS) $O_{A}X_{A}Y_{A}Z_{A}$ to provide the high accuracy spatial position information of each marker; the camera (CCS) $O_{C}X_{C}Y_{C}Z_{C}$ and PCS ouv (defined in Section 2), and the machine coordinate system (MCS) $O_{M}X_{M}Y_{M}Z_{M}$ to represent the contouring error of a movement trajectory. The origin of the ACS is fixed on the artifact, with its origin $O_{A}$ located in the central point of the upper-right marker. The positive $X_{A}$- and $Y_{A}$-axes point left and downward, respectively; while the positive $Z_{A}$-axis points outside. Before the machine movement, the MCS is established and its origin coincides with that of ACS. Below we take the contouring error detection of a spatial contour (Section 5.1) as an example to describe the 3D high temporal-spatial measurement method in detail:

1) System setup and imaging parameters adjustment

**Figure 16:**
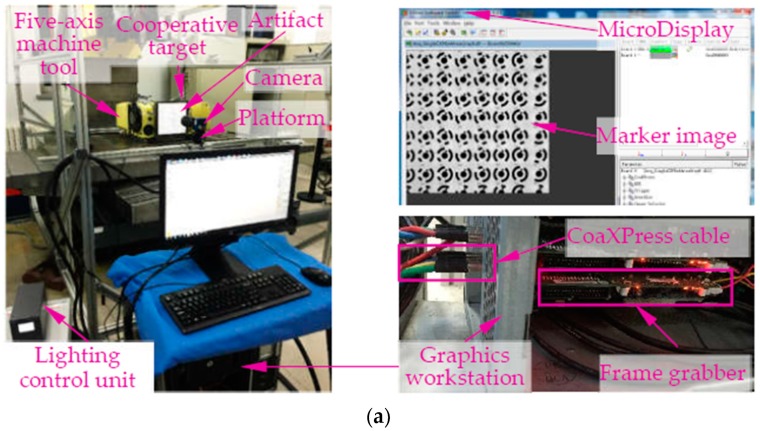

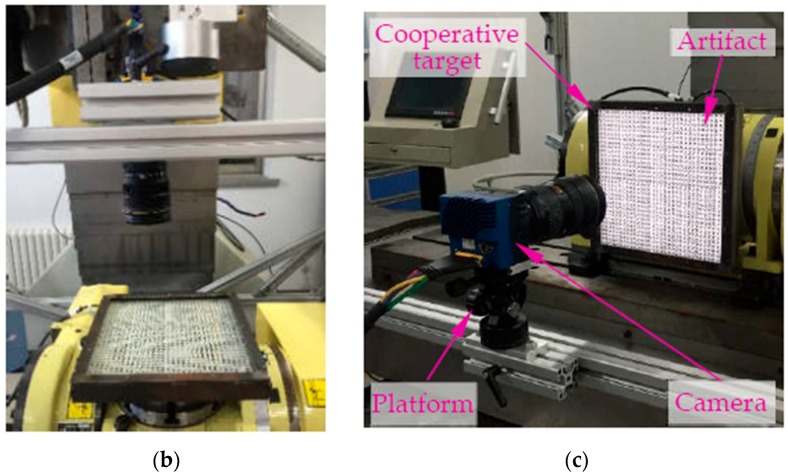
Experimental system and test configurations. (**a**) Experimental system. (**b**) Test configuration for butterfly curve. (**c**) Test configuration for spatial curve.

The vision measurement system is installed on the platform outside the machine (Figure 16c) to avoid vibrations. Considering the Z-direction variation (about 55 mm) of the spatial contour, the FOV and camera resolution are set to 60 × 60 mm and 3072 × 3072 pixels, respectively. Then, with the 0.03–0.05 subpixel accuracy of the gray centroid algorithm [48], an ideal measurement accuracy of 2 μm can be obtained with a 50 mm focal length at focusing distance of 450 mm. Furthermore, under the imaging parameters, more than 9 markers can appear in each image frame to guarantee the feasibility of the PNP algorithm.

2) Camera calibration

Since the control field (Figure 3) and the artifact (Figure 6a) can be exchanged in the designed cooperative target. Therefore, before the measurement, the cooperative target with the control field is first placed on the worktable. Then, combining with the coded markers, the camera pose is adjusted by the PNP algorithm. In the process, the intrinsic matrix K in Equation (1) are repeatedly calibrated using Zhang’s method [40], until the optical axis is perpendicular to the control field. Thereafter, the proposed camera calibration method considering the distortion partition of DOF in Section 2.2 is performed. The specific process and accuracy verification is detailed in Section 5.2.

3) 3D contouring error detection of wide range trajectories by using PNP algorithm

The coded marker $P^{r}$, i.e., the origin $O_{A}$ of the ACS, is used as the ‘reference feature’ to represent the entire movement trajectory. Since the contouring error is expressed in the MCS (ISO 10791-6), hence, before measurement, data transformation matrix between VCS and MCS is determined by the method similar to our previous work [15], which can be given by:

(11) $$\{\begin{array}{c} \frac{x_{i}-{}^{C}X_{M}}{m_{x}}=\frac{y_{i}-{}^{C}Y_{M}}{n_{x}}=\frac{z_{i}-{}^{C}Z_{M}}{p_{x}}\\ \frac{x_{i}^{\prime }-{}^{C}X_{M}}{m_{y}}=\frac{y_{i}^{\prime }-{}^{C}Y_{M}}{n_{y}}=\frac{z_{i}^{\prime }-{}^{C}Z_{M}}{p_{y}}\\ \left(\begin{array}{cccc} {}^{M}X & {}^{M}Y & {}^{M}Z & 1 \end{array})=M_{C}^{M}\cdot (\begin{array}{cccc} {}^{C}X & {}^{C}Y & {}^{C}Z & 1 \end{array}\right) \end{array}$$

where ${}^{C}O_{M}(\begin{array}{ccc} {}^{C}X_{M} & {}^{C}Y_{M} & {}^{C}Z_{M} \end{array})$ are the 3D coordinates of point $P^{r}$ in CCS when the CNC machine tool is zeroed. In this position, the MCS is established. $\left(\begin{array}{ccc} x_{i} & y_{i} & z_{i} \end{array}\right)$ and $\left(\begin{array}{ccc} x_{i}^{\prime } & y_{i}^{\prime } & z_{i}^{\prime } \end{array}\right)$ depicts the 3D coordinates of the point $P^{r}$ in CCS when the worktable (i.e., artifact) is moved to the *i*th position along X- and Y-axis. Based on position data of $P^{r}$ in CCS, the direction vector of X- and Y-axis of MCS, i.e., $\left(\begin{array}{ccc} m_{x} & n_{x} & p_{x} \end{array}\right)$ and $\left(\begin{array}{ccc} m_{y} & n_{y} & p_{y} \end{array}\right)$, can be fitted by the least square method, and that of Z axis can be obtained by the right hand rule. $M_{C}^{M}$ represents the transformation matrix from the CCS to the MCS, including the rotation matrix $R_{CM}$ and the translation matrix $T_{CM}$. The main difference is that the 3D coordinates calculation of point $P^{r}$ in this paper is constructed by PNP algorithm, while in [15], it is calculated by triangulation.

During measurement, the CCS and MCS are fixed, while the ACS makes interpolation motion with the worktable. Simultaneously, the time-varying markers are continuously imaged on the camera. As described in step 1, the maximum frame rate can reach to 208 FPS by reducing both the camera resolution and the FOV. While for enlarging the measurable range of the detection system in condition of small FOV, we use a priori information among coded markers on the artifact to deduce the 3D position of the ‘reference feature’ $P^{r}$. As discussed above, the measurable speed is enlarged by sacrificing FOV, then the small FOV will lead to the invisibility of the ‘reference feature’ in some images. In this case, together with the pre-calibrated position relationship between the visible marker and the ‘reference feature’, the visible auxiliary coded markers are used by the OPNP algorithm to deduce the 3D position of the ‘reference feature’. Suppose that a total of Q frames are acquired, for the ith frame, to calculate the 3D coordinates of $P_{i}^{r}$, firstly, the number of j complete coded markers, denoted by $p_{i}^{1}(\begin{array}{cc} u_{i}^{1} & v_{i}^{1} \end{array})$, $p_{i}^{2}(\begin{array}{cc} u_{i}^{2} & v_{i}^{2} \end{array})$, $p_{i}^{3}(\begin{array}{cc} u_{i}^{3} & v_{i}^{3} \end{array})$ ⋯ $p_{i}^{j}(\begin{array}{cc} u_{i}^{j} & v_{i}^{j} \end{array})$, are identified and located using the image processing method proposed in Section 3.2. Let the corresponding points in ACS are ${}^{A}P_{i}^{1}(\begin{array}{ccc} {}^{A}X_{i}^{1} & {}^{A}Y_{i}^{1} & {}^{A}Z_{i}^{1} \end{array})$, ${}^{A}P_{i}^{2}(\begin{array}{ccc} {}^{A}X_{i}^{2} & {}^{A}Y_{i}^{2} & {}^{A}Z_{i}^{2} \end{array})$, ${}^{A}P_{i}^{3}(\begin{array}{ccc} {}^{A}X_{i}^{3} & {}^{A}Y_{i}^{3} & {}^{A}Z_{i}^{3} \end{array})$ ⋯ ${}^{A}P_{i}^{j}(\begin{array}{ccc} {}^{A}X_{i}^{j} & {}^{A}Y_{i}^{j} & {}^{A}Z_{i}^{j} \end{array})$. Then, using the pre-calibrated camera parameters, the 3D point ${}^{C}P_{i}^{r}$ in CCS can be solved by OPNP algorithm, and the measured point in MCS ${}^{M}P_{i}^{r}$ can be calculated by datum transformation in Equation (11). Finally, the 3D contour $L_{r}$ can be calculated by traversing all the images, and the contouring error E can be deduced by comparing the difference between measured trajectory and the nominal one $E=L_{r}-L_{m}$. In this way, the measurable range no longer depends on the size of the FOV, but on the size of the artifact, and the two indicators can thus be simultaneously increased.

## 5. Contouring Error Detection Test and Vision Measurement Accuracy Verification

### 5.1. Experimental Equipment and Tested Trajectories

As shown in Figure 16, the experimental equipment includes a five-axis CNC machine tool, a camera, a platform, a graphics workstation, a frame grabber and a cooperative target. As hardware platform, an EoSens^®^ 25CXP CMOS camera with full-resolution of 5120 × 5120 pixels is selected. The camera is connected to a microEnable 5 frame grabber (VQ8-CXP6D) within a 22’’ Windows XP-based workstation by CoaXPress cable. For the software library, GenICam standard is used to configure and trigger the camera, and images are visualized by the microDisplay software. All algorithms are developed with the aid of the machine vision toolbox of MATLAB. In terms of the synchronous trigger between the camera and the machine tool, it is not so strict for the monocular vision system. We only need to ensure that the whole movement trajectory can be recorded. Thus, the software trigger is first used to allocate memory for image acquisition, thereafter the machine tool is triggered manually to perform the trajectory interpolation. In practical measurement, the FOV and camera resolution are set to 60 × 60 mm and 3072 × 3072 pixels, respectively. Though, the resultant frame rate can be increased up to 208 FPS, the sufficient frame rate of 100 FPS is selected to acquire high-quality marker images. Then, with the 0.03–0.05 subpixel accuracy of the gray centroid algorithm [48], an ideal measurement accuracy of 2 μm can be obtained with a 50 mm focal length at focusing distance of 450 mm. The aperture is set at f/22 to ensure that the relatively large DOF of 71 mm can accommodate the distance change (about 55 mm) of the spatial contour. Besides, large artifact with size of 231 mm × 231 mm is designed to increase the measurable range of the system. Other experimental parameters are shown in Table 1.

Clearly, the large-scale measurement capability is guaranteed by the pre-calibrated large-size artifact. However, the measurement advantage of speed depends entirely on whether non-fuzzy images can be acquired at the set frame rate. Thus, performance test is conducted to verify the relatively high feed measurement capacity. As shown in Table 2, we reduced the camera resolution from 5120 × 5120 pixels to 3072 × 3072 pixels and 1024 × 1024 pixels by the built-in ROI function. Correspondingly, three experimental frame rates are obtained: 25 FPS, 100 FPS and 150 FPS.

**Figure 17:**
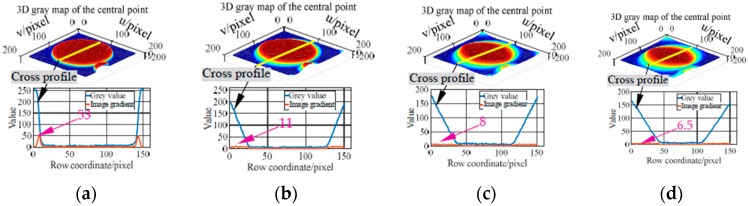
3D grey map and grey gradient of the central point captured at 25 FPS with 5120 × 5120 pixels. (**a**) Results of a static image (ground truth). (**b**) Results at 3 m/min. (**c**) Results at 5 m/min. (**d**) Results at 7 m/min.

**Figure 18:**
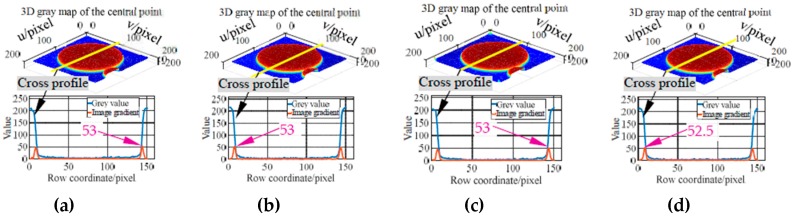
3D grey map and grey gradient of the central point captured at 100 FPS with 3072 × 3072 pixels. (**a**) Results of a static image (ground truth). (**b**) Results at 3 m/min. (**c**) Results at 5 m/min. (**d**) Results at 7 m/min.

**Figure 19:**
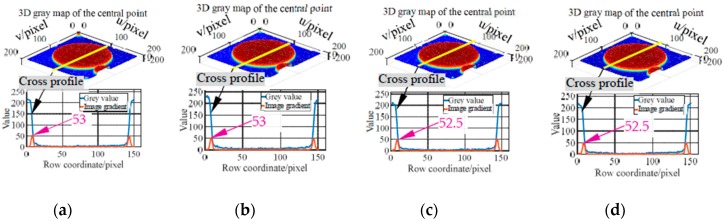
3D grey map and grey gradient of the central point captured at 150 FPS with 1024 × 1024 pixels. (**a**) Results of a static image (ground truth). (**b**) Results at 3 m/min. (**c**) Results at 5 m/min. (**d**) Results at 7 m/min.

Based on the above three types of imaging parameters, images of the markers moving along with the *X* axis of the machine are acquired (measurement configuration is shown in Figure 16b). In the tests, the feed rates are set to 3 m/min, 5 m/min and 7 m/min, respectively. Subsequently, the grey characteristics of the marker with the code value of 397 are studied. And the 3D grey maps captured at different feed rates using imaging parameters in the three columns of Table 2 are plotted, as well as the cross-section grey curve passing the point center (i.e., Figure 17, Figure 18 and Figure 19). Taking the captured static image as a reference, the sharpness of the point edge at different feed rates is measured by comparing the image gradient with that of a static image (i.e., Figure 17a, Figure 18a, and Figure 19a). As can be seen from the 3D grey maps in Figure 17b–d, when using the same frame rate (i.e., 25 FPS) as in [15], images of central point are obviously degraded. In addition, at 3 m/min, 5 m/min and 7 m/min, the image gradients of the point edge are 11, 8 and 6.5, far less than the reference value of 53. The larger the feed rate, the smoother the point edge. However, the results obtained by the other two imaging parameters (i.e., Figure 18 and Figure 19) show satisfactory results. The 3D grey maps captured at different feed rates (i.e., Figure 18b or Figure 19b) has a good consistency with the reference (i.e., Figure 18a or Figure 19a). Moreover, the image gradient values of the point edge at different feed rates under the two imaging conditions differs from the reference value by about 0.5 pixel, indicating the sharpness of the point edge. Additionally, as illustrated in Table 2, by reducing the camera resolution, the maximum frame rate is increased from 33 FPS to 208 FPS and 308 FPS, respectively, which verifies the feasibility of the proposed method in measuring relatively high-dynamic contouring error.

**Figure 20:**
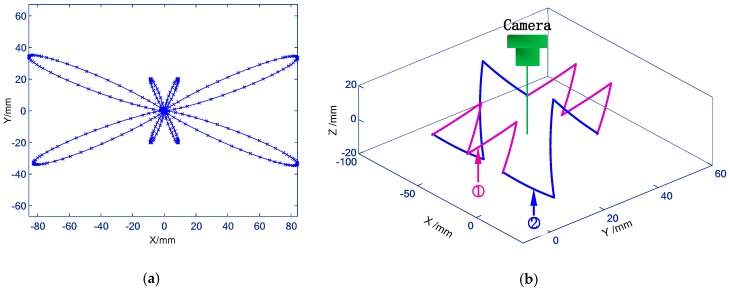
Commanded paths in MCS. (**a**) Large-scale butterfly curve in MCS. (**b**) Commanded spatial curve in MCS.

To verify the advantages of the proposed knowledge-driven contouring error detection approach in multi-dimensional, high speed, wide working range as well as the various forms of trajectories measurement over the existing vision and non-vision methods. Contouring errors of two types of paths, i.e., planar contour and spatial trajectory, are evaluated. The former is the wide range butterfly curve (Figure 20a) which is interpolated by the predetermined *X*- and *Y*-axis, of which the polar equation can be expressed as $r=6\cdot e^{\left(\cos(2\theta )-2\cos(8\theta )+(\sin{\left(\theta /6\right)}^{5})\right)}$ ($X\in \left[\begin{array}{cc} 85.601\mathrm{mm} & 84.132\mathrm{mm} \end{array}\right]$ and $Y\in \left[\begin{array}{cc} 34.554\mathrm{mm} & 35.138\mathrm{mm} \end{array}\right]$), the working range in *X*-axis is twice that of our previous work [15], and about 2.8 times of the FOV selected in this paper. Another is the spatial path shown in Figure 20b, in which the whole curve can be derived by the offset or rotation of the two paths: $\{\begin{array}{c} x_{1}=-l_{c}\cdot \sin\theta_{c}+l_{c}\cdot \sin\beta_{c}\\ y_{1}=315\cdot (\theta_{c}-\beta_{c})/4\pi \\ z_{1}=l_{c}\cdot \cos\theta_{c}-l_{c}\cdot \cos\beta_{c}\\ \theta_{c}\in \left[\begin{array}{cc} \beta_{c} & \beta_{c}+\pi /9 \end{array}\right] \end{array}$ and $\{\begin{array}{c} x_{2}=-270\cdot (\theta_{a}+\beta_{a})/\pi \\ y_{2}=-l_{a}\cdot \sin\theta_{a}-h\\ z_{2}=l_{a}\cdot \cos\theta_{a}+l_{a}\cdot \cos(\pi -\beta_{a})\\ \theta_{a}\in \left[\begin{array}{cc} -\beta_{a} & -\beta_{a}+\pi /18 \end{array}\right] \end{array}$, where $\beta_{a}=\mathrm{at}\;\mathrm{an}(l_{c}\cdot \sin\beta_{c}/h)$ and $l_{a}=\sqrt{l_{c}^{2}\sin^{2}(\beta_{c})+h^{2}}$; h, $l_{c}$ and $\beta_{c}$ depend on the position of markers. To achieve contouring error measurement, considering the defocus effect caused by the small DOF under imaging parameters of large focal length and small object distance, two measurement configurations are used. That is, for setup 1 (Figure 16b), the camera is placed above the XOY plane to measure contouring error of the butterfly curve, while setup 2 (Figure 16c) with camera mounting in front of the machine tool is utilized to detect motion error of the spatial trajectory.

### 5.2. Experiment for Verifying the Proposed Calibration Method

**Figure 21:**
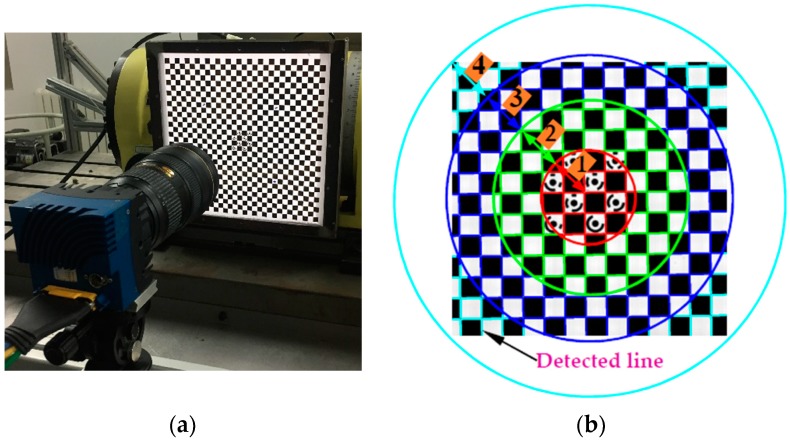

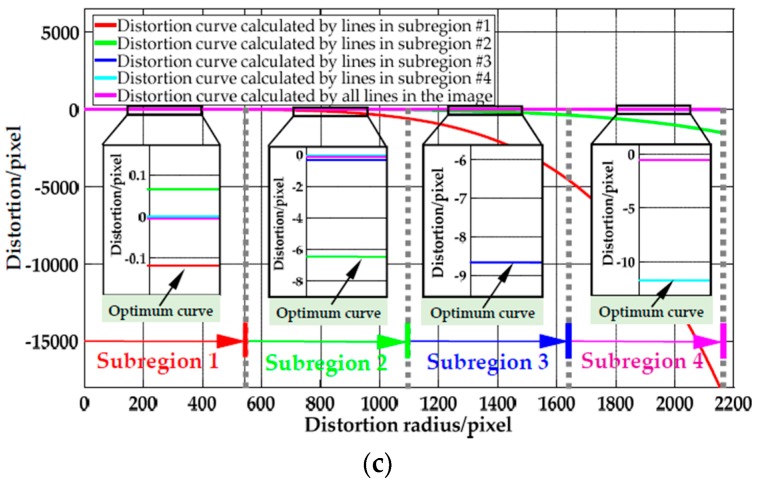
Distortion curves. (**a**) Experimental setup. (**b**) Extracted straight lines on the four subregions of the front plane image by equal-radius partition. (**c**) Distortion curves of the front plane calculated by minimizing straightness errors.

Before conducting contouring error detection experiment, we verify the measurement accuracy of the distortion calibration method proposed in Section 2.2. As shown in the Figure 21a, after the alignment, the control field is driven by the machine to move four positions within the DOF perpendicular to the optical axis, two object planes at the front and rear position of the DOF are used as the reference to estimate the distortion behavior of the other two middle planes.

For a clearer description, the verification process is divided into two steps:

1) Accuracy verification of the 3D distortion partition model

As shown in the Figure 21a, after the alignment, the control field is driven by the machine to move four positions within the DOF perpendicular to the optical axis. Two object planes at the front and rear position of the DOF are used as the reference to estimate the distortion behavior of the other two middle planes:

(1) Accuracy verification of equal-radius partition model
  As shown in Figure 21b,c, distortion curve of each subregion is different from that of calculated by all the lines in the image. Firstly, the performance of in-plane distortion partition model is judged by the straightness error after distortion correction. As illustrated in Table 3, the maximum and average straightness errors of each subregion are smaller than that are calculated by all the lines in the image, which indicate the accuracy of the proposed partition method. The optimal distortion curve for each subregion can be seen in the enlarged view of Figure 21c.
(2) Accuracy verification of the 3D distortion partition model
  Then, based on the front and rear object planes with known depths and the calibrated distortion parameters, the distortion coefficients on each partition of the two middle object planes are estimated by the method in Section 2.2. Thereafter, the derived distortions of the two middle planes are compared with that calculated directly by the plumb-line method to verify the accuracy of the proposed DOF distortion partition model.
  Table 4 illustrates the difference |*C* − *O*| between the distortion calculated with or without DOF distortion partition model and the observed one. The results indicate that the maximum and average differences are 1.75 μm and 0.86 μm, while the distortion differences calculated without the partition model are more than twice the corresponding difference calculated with the partition distortion, which show the high accuracy of the proposed partition method.

2) Accuracy verification of camera calibration

**Figure 22:**
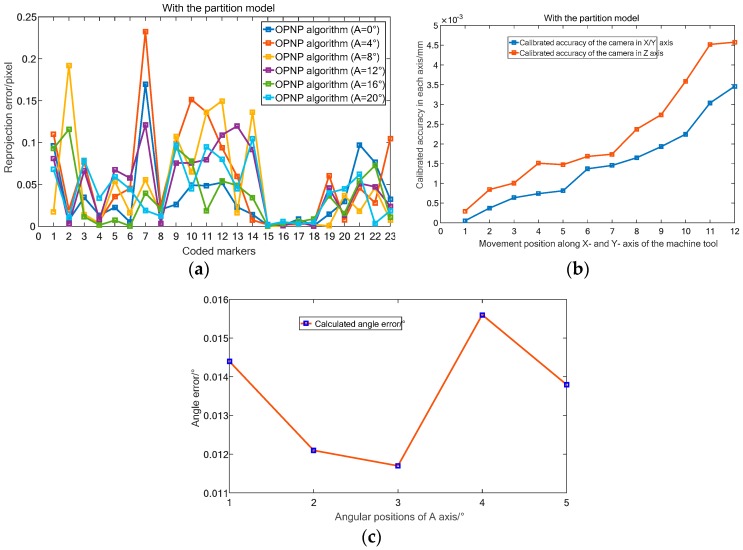
Accuracy verification of the camera calibration method. (**a**) Reprojection error of OPNP algorithm with partition model. (**b**) Calibrated accuracy of the camera in each axis. (**c**) 3D accuracy verification results of the vision system.

To verify the calibration accuracy, the DOF distortion partition model is embedded in the selected OPNP algorithm to correct the pixel position deviation. Similar to the experimental equipment (Figure 14a) and the verification process in Section 4.1, we first verify the calibration accuracy by using the reprojection error in pixel. As illustrated in Figure 22a, the maximum and average reprojection errors of the six angle positions using the OPNP algorithm with partition model are 0.23 pixel and 0.04 pixel, respectively. Additionally, the calibrated accuracy of the camera on each axis is validated. To perform camera calibration, the image plane should be installed parallel to the control field. Since the CMOS sensor has the same pixel size and imaging resolution in horizontal and vertical directions, we assume that the camera has the same measurement accuracy in the two directions. In practical application, the control field is driven to move 13 positions with an interval of 3 mm in the *X*- and *Z*-axis of the camera. Then, considering the high accuracy of machine tool static positioning, the vision calibrated accuracy is evaluated by the distance deviation of two adjacent stop positions. As shown in Figure 22b, the maximum and mean calibrated accuracy of the camera in *X*/*Y*- and *Z*-axis are 3.4 µm/1.6 µm and 4.5 µm/1.4 µm, while the standard deviations are 1.0 µm and 1.6 µm, respectively. The results indicate the high calibrated accuracy of the camera in three axes.

Besides, we apply the algorithm to construct the angle between two adjacent angular positions, and validate the measurement accuracy of the system by comparing it with the actual motion angle. The accuracy verification results in Figure 22c illustrate that the maximum and mean angle errors are 0.0157° and 0.0132°, which verifies the 3D measurement accuracy of the vision system.

### 5.3. Case Study for Illustrating Advantages in 3D High Temporal-Spatial Measurement

In our previous work [15], due to the blurring effect caused by the fast movement of the target, the maximum measurable traverse speed was limited by 2 m/min. To demonstrate the capacity in synchronously extending the measurable range and speed, as well as of the 3D measurement of the contouring error. The contouring error of a large-scale butterfly curve (Figure 20a) is tested at 3 m/min and 5 m/min, respectively.

**Figure 23:**
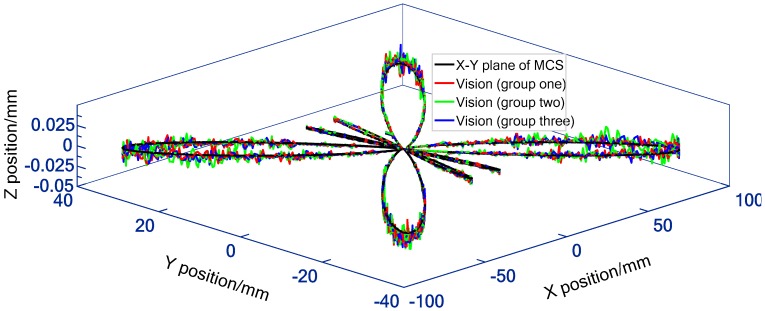
3D large-scale butterfly path expressed in MCS by data transferring (3 m/min).

**Figure 24:**
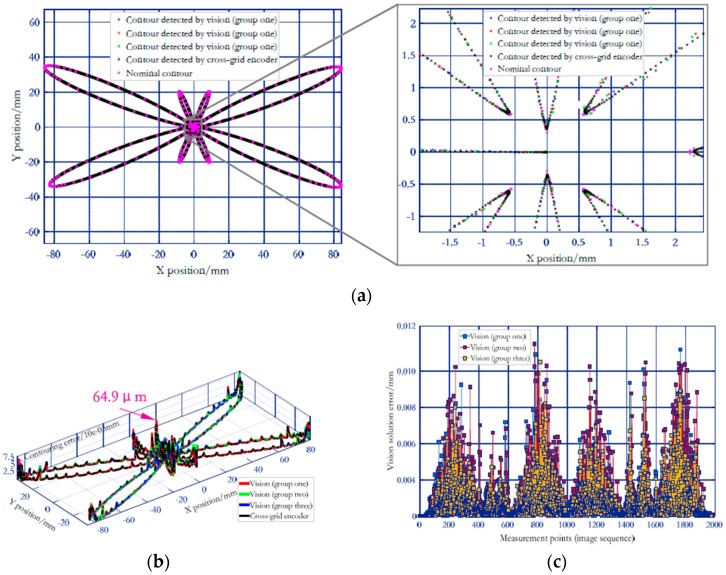
Verification results of vision system in solving contouring error of large-scale butterfly path (3 m/min). (**a**) 2D path detected by the two devices. (**b**) Contouring error obtained by the two devices. (**c**) Verification results of vision system.

**Figure 25:**
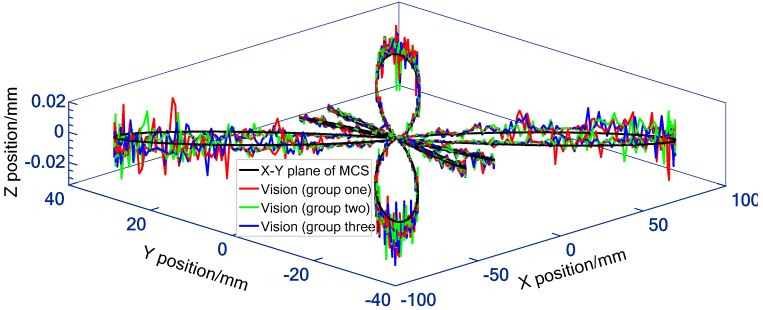
3D large-scale butterfly path expressed in MCS by data transferring (5 m/min).

**Figure 26:**
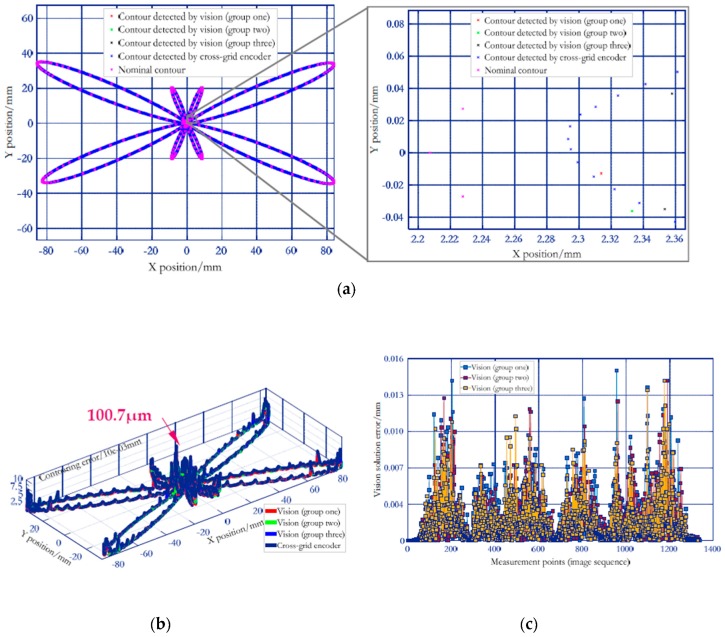
Verification results of vision system in solving contouring error of large-scale butterfly path (5 m/min). (**a**) 2D path detected by the two devices. (**b**) Contouring error obtained by the two devices. (**c**) Verification results of vision system.

In practical application, the experimental process was repeated three groups for each feed rate, and the three repetition results are plotted on one graph (e.g., Figure 23). Since the interpolation motion is triggered manually, the sampling positions of the camera on the machine tool movement among the three groups are different (e.g., Figure 24c). This is equivalent to increasing the sampling points of motion trajectory, so that the estimation of contouring error is more sufficient. Besides, the number of sampling points varies with the feed rate. For each group of experiments at 3 m/min and 5 m/min, a total of about 2000 (e.g., Figure 23 and Figure 24c) and 1350 (e.g., Figure 25 and Figure 26c) image frames are collected, respectively. This is mainly due to the fact that the faster the interpolation speed is, the smaller the total duration. Figure 23 and Figure 25 depict the 3D large-scale trajectories constructed in the MCS at two different traverse speed using the single camera. The results indicate that the method enable the large-scale path measurement in small FOV. Meanwhile, the 3D measured paths with time-varying curvature validate that the vision method breaks the limitations of the measurable dimension and trajectories of some existing equipment (e.g., ball-bar, cross-grid encoder and R test). As illustrated in Figure 23 and Figure 25, the paths interpolated by the predetermined *X*- and *Y*-axis float above and below the movement plane. The maximum float range can reach 23.2 μm and 29.1 μm, respectively, which is mainly caused by the defects of the numerical control system and the machine structure (e.g., vibration and straightness error).

**Figure 27:**
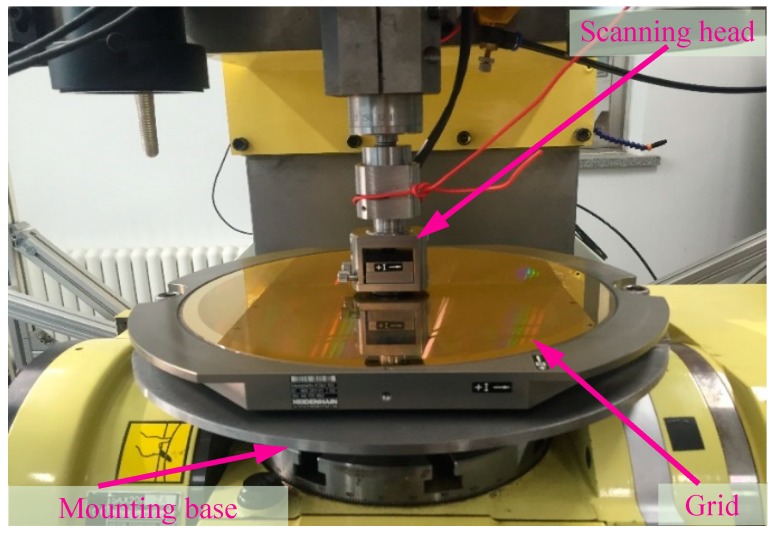
Test configuration of the cross-grid encoder.

Additionally, as described in [15], the commercial cross-grid encoder (Figure 27) with high resolution of 0.5 μm is employed to measure the butterfly curve under the same conditions (test configuration in Figure 27 is the same as in Figure 16b). Firstly, the 3D visual measured points are projected onto the XY plane of machine tool coordinate system to obtain the 2D contour. At the same time, the 2D interpolated butterfly contour is measured by cross-grid encoder under the same conditions. Then, according to ISO 10791-6, 2D contours measured by the two devices are compared with the nominal ones (commanded path) to obtain the contouring error. Thereafter, the 2D contour measured by cross-grid encoder is considered as the standard, and the accuracy of the vision method in detecting contouring error of butterfly curve is verified by comparing the difference between the two trajectories measured by the camera and the cross grid encoder. Figure 24a and Figure 26a indicate that the 2D paths detected by both the vision and cross-grid encoder returns a consistent trend with the nominal ones. Figure 24b and Figure 26b describe the 2D contouring error of the trajectories measured at two feed rates by the two means, while the Figure 24c and Figure 26c depict the verification results of vision measurement accuracy. To give a better assessment of both the contouring error and vision measurement accuracy, a total of 8000 points are sampled by the cross-grid encoder on the whole motion trajectory. For contouring error of the butterfly path detected by cross-grid encoder at 3 m/min and 5 m/min, the maximum and mean contouring error are 64.9 μm/13.2 μm (at 3 m/min, Figure 24b) and 100.7 μm/15.7 μm (at 5 m/min, see Figure 26b), respectively. As illustrated in the two figures, the higher the feed rate, the larger the contouring error, and the maximum contouring error appears in the contour with large curvature. The difference between the maximum and minimum contouring errors at 3 m/min can be up to 56.6 μm, while at 5 m/min, it is up to 89.2 μm. The verification data are presented in Figure 24c and Figure 26c, compared to the paths detected by the cross-grid encoder, the results show that the vision-based maximum and mean solution error at two different feed rates are 11.3 μm/3.4 μm (at 3 m/min, Figure 24c) and 14.1 μm/3.9 μm (at 5 m/min, Figure 26c), both are less than 1/3 of the error to be solved. The results demonstrate that the 3D high temporal-spatial measurement method enables the vision to have both wide range and relatively high-dynamic measurement capabilities. Besides, the standard deviations of the vision measurement accuracy at 3 m/min and 5 m/min are 1.4 μm and 1.7 μm respectively, which shows the good stability of the measurement accuracy.

### 5.4. Case Study for Highlighting 3D Detection of Contouring Error of a Space Trajectory

As mentioned in Section 5.2, we verified the accuracy of the proposed DOF distortion partition method, as well as the 3D measurement accuracy. On this basis, to further illustrate the advantages of the proposed monocular vision scheme in spatial contouring error detection over the existing equipment (e.g., telescoping ballbar and cross-grid encoder). The contouring performance of the interpolated spatial curve shown in Figure 20b is assessed at 3 m/min.

**Figure 28:**
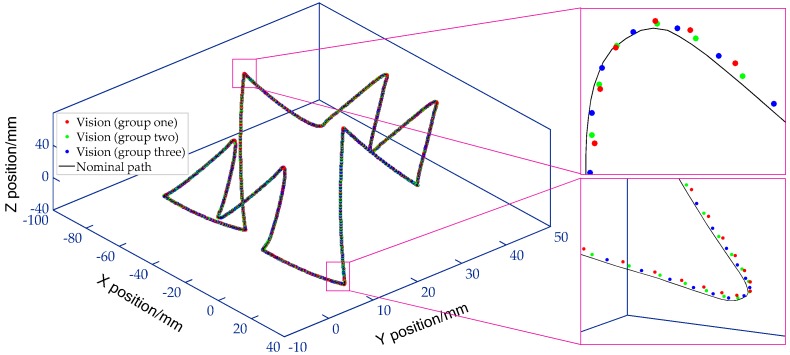
3D large-scale butterfly path expressed in MCS by data transferring (3 m/min).

To improve the vision positioning accuracy of fiducial markers, in practical measurement, the PNP algorithm is first used to estimate the 3D position of point *O_G_* on the artifact. Then, the four subregions-based distortion partition in the object plane perpendicular to the optical axis is extended to the DOF. Thereafter, the spatial coordinates of point *O_G_* is re-corrected to a precise solution using the proposed subregion based distortion model. The measured 3D contour of the spatial path is shown in the Figure 28, in which scatter points with red, green and blue marks are the three groups of movement paths measured by monocular vision. While the black ones form the nominal curve. For each experimental group, 2500 points on the trajectory are sampled by the vision method. As can be seen from the enlarged view, the three repetition results have good consistency in reflecting the trend of contouring error. For place where the curvature changes rapidly, large contouring errors induced by the servo mismatch are more likely to occur. The difference between the maximum and minimum contouring errors can be up to 72 μm. We performed statistical analysis on the measured data, and the results reveal that maximum and mean contouring error caused by the imperfect machine tool are 78.9 μm and 11.7 μm. Additionally, the standard deviation of contouring error is 9.3 μm, which indicates that the machine has a stable and small contouring error in most contour positions of the in contrast to where the curvature changes drastically. This case study highlights the measurement capability of the vision metrology in detecting contouring performance of a space trajectory.

Though contouring error of a spatial path can be detected. In fact, the camera focusing system is probably not capable of following target changes which involve large change in distance. This is mainly caused by the relatively small DOF. The key indicators that affect DOF are working distance, focal length and aperture. Although the DOF of a single camera is greater than that of binocular camera, both of the two systems inevitably use small working distance and large focal length in high-accuracy applications. Consequently, the small DOF induced defocus blur degrades the image of coded markers, which significantly decreases the vision measurement accuracy. Since DOF is independent of FOV and camera resolution, thus, the proposed method cannot further enhance the DOF.

### 5.5. Remarks on Major Contributors for Measurement Uncertainties

The issue of measurement has been explored in prior studies [52]. We reanalyze all the links in the vision measurement process, and major contributors affecting the vision measurement uncertainty are presented, which are detailed as follows:

1) Error of a priori information

To achieve vision measurement with the proposed vision method, artifact calibration is necessary. In this paper, we calibrate the 3D position of the coded markers (i.e., a priori information) using high-accuracy commercialized instrument HEXAGON OPTIV reference (Measurement uncertainty $E_{x}$, $E_{y}$: (0.25 + *L*/900) μm and $E_{z}$: 0.5 μm), where L is the measurement length (in mm). On the one hand, PNP measurements are valid when more than three markers with known spatial position in FOV. On the other hand, when performing wide range measurement, the 3D position of the invisible ‘reference feature’ is derived based on a priori information. Therefore, the markers’ position calibration error contributes the vision measurement uncertainty throughout the measurement.

2) Image size of the marker

In this paper, the grey centroid method is applied to locate the center (*x_M_* *y_M_*) of the marker on the image, which can be described by the classic equation:

(12) $$\{\begin{array}{c} x_{M}=\sum_{i=1}^{n}\left(x_{i}\cdot t\cdot s_{i}\right)/\sum_{i=1}^{n}\left(t\cdot s_{i}\right)\\ y_{M}=\sum_{i=1}^{n}\left(y_{i}\cdot t\cdot s_{i}\right)/\sum_{i=1}^{n}\left(t\cdot s_{i}\right) \end{array}$$

where n denotes the number of pixels to be processed, $s_{i}$ represents the grey value at pixel position $\left(\begin{array}{cc} x_{i} & y_{i} \end{array}\right)$. According to whether the pixel position participates in the calculation, t is set to 0 or 1. Then, do error propagation to Equation (12) to determine theoretical accuracy:

(13) $$\{\begin{array}{c} \sigma_{x_{M}}=\sigma_{s}\cdot \sqrt{\sum {\left(x_{i}-x_{M}\right)}^{2}}/\sum s_{i}\\ \sigma_{y_{M}}=\sigma_{s}\cdot \sqrt{\sum {\left(y_{i}-y_{M}\right)}^{2}}/\sum s_{i} \end{array}$$

As illustrated in Equation (13), the standard deviation of centroid is clearly positively correlated with the grayscale noise (i.e., $\sigma_{s}$) and the marker image size. For instance, for a marker occupying 6 pixels whose grey value is 220, the theoretical standard deviation of the centroid is about $\sigma_{x_{M}}=\sigma_{y_{M}}=\;0.005\mathrm{pixel}$ when image grey value noise is 0.6. Besides, according to [48], image size of marker also influence the center deviation caused by the perspective effect. The larger the image size occupied, the larger the eccentricity. Through the above analysis, the target size is also the main factor influencing the measurement uncertainty. While this issue was not studied in our experimentations. For high-accuracy applications (smaller than 0.5 μm), the image size of the target should be as small as possible, but at least 5 pixels for anti-noise and algorithm application.

3) Alignment error

**Figure 29:**
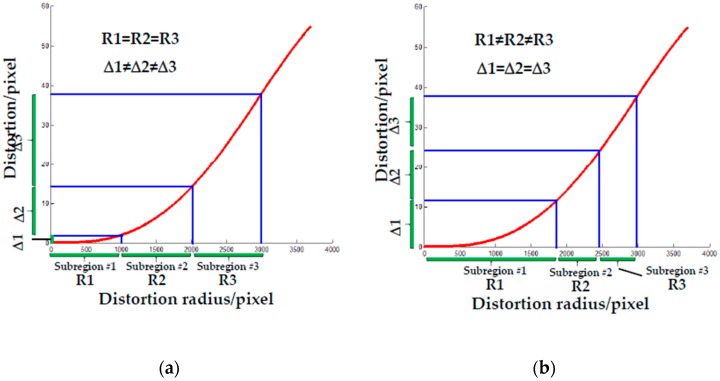
Distortion partitioning method. (**a**) Equal radius partition. (**b**) Equal distortion partition.

In this paper, a camera calibration method considering the distortion partition of the DOF is proposed, which allows the calculation of distortion coefficients of arbitrary object distance by two known object planes (Equation (6)). Therefore, before using the proposed method (Section 2.2) to calibrate camera parameters, the image plane should be installed parallel to the control field. In practice, combining with the Zhang’s method [40], it is repeatedly adjusted by the PNP algorithm. And the alignment error can only be controlled within 0.1°, reducing the subsequent camera calibration accuracy. Additionally, the distortion coefficients describe the goodness-of-fit of the distortion in each subregion by minimizing the straightness error. For each region with equal partition radius R1 = R2 = R3 (Figure 29a), according to Equation (2), the calculated accuracy of distortion coefficients varies with different distortions Δ1 ≠ Δ2 ≠ Δ3. And the larger the distortion variation, the lower the estimation accuracy of distortion (see in Table 3). Thus, we assume that if the distortion in the object plane is partitioned by equal-distortion criterion (Figure 29b), i.e., Δ1 = Δ2 = Δ3. Then, although the partition radiuses are different R1 ≠ R2 ≠ R3, we can achieve high accuracy of distortion coefficient calculation. Our further study will focus on it.

## 6. Conclusions

In this paper, a 3D high temporal-spatial measurement method and system based on a single camera are proposed, this knowledge-driven approach realizes the 3D detection of contouring errors of arbitrary paths, especially that of interpolated spatial contours. The innovations of this paper are the work to improve the accuracy, efficiency and ability (i.e., measurable speed and working range) of the vision measurement, which is detailed as follows: a camera calibration method considering the distortion partition of the FOV is proposed, which solves the problem that the DOF-dependent imaging distortion seriously restricts the vision measurement accuracy; both a new encoding method and the decoding method based on finding the optimal start bit are proposed, which improve the marker identification efficiency in image processing. Finally, together with a priori information, the 3D measurement of large-scale contouring error under relatively high dynamic conditions is realized by the PNP algorithm. After performing the performance test of the vision system, contouring errors of both a planar and a spatial trajectory are measured in the laboratory. The statistical analysis results verify the measurement ability of proposed monocular vision-based method in multi-dimensional (versus double ballbar and cross-grid encoder), wide working range (versus R-test) and various forms of trajectories (versus double ballbar, R-test and cross-grid encoder). Finally, other factors affecting the uncertainty of the vision measurement are analyzed. This technique has potential applications in enhancing the dynamic behavior of low-accuracy CNC machines. The main limitation of the research is the relatively low measurement accuracy compared to binocular vision, as well as the inability to measure trajectories with relatively large variations in DOF. Therefore, our next objective focuses on the accuracy improvement of PNP algorithm, as well as the extension of the DOF of the vision system.

## Figures and Tables

**Table 1:** Experimental parameters.

Index	Parameter Values
Camera and resolution	Camera: EoSens^®^ 25 CXP; Full resolution: 5120 × 5120 pixels
Lens	Nikon 24–70 mm
Lens mount	F-Mount
Exposure time	3000 μs
Spatial resolution (without subpixel accuracy)	0.0195 mm/pixel
Size of the measurement basis	231 mm × 231 mm
FOV	60 mm × 60 mm
Light source	Flat backlight
Light-emitting area	250 mm × 250 mm
Number of coding primitives	1024 (see Figure 6a for detail)
Geometrical accuracy of single coded targets	<1 μm
Calibration accuracy of spatial geometric information	0.5 μm

**Table 2:** Frame rate and markers’ image obtained by decreasing the camera resolution.

Camera Resolution	5120 × 5120 pixels	3072 × 3072 pixels	1024 × 1024 pixels
The collected static image	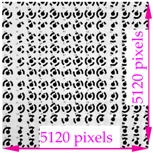	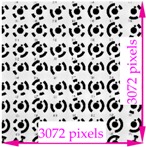	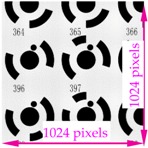
Allowable maximum FPS	33 FPS	208 FPS	308 FPS
FPS used in tests	25 FPS	100 FPS	150 FPS

**Table 3:** Accuracy verification of the in-plane distortion partition model.

	Subregion 1	Subregion 2	Subregion 3	Subregion 4	Entire Image
Maximum distance error/pixel	0.12	0.19	0.45	0.81	3.53
Mean distance error/pixel	0.07	0.06	0.11	0.24	0.27

**Table 4:** Accuracy verification of the proposed DOF distortion partition model.

Object Plane /mm	Subregion	Distortion				
		Observed (μm)	With Partition Model		Without Partition Model	
			Calculated (μm)	Difference |*C* − *O*| (μm)	Calculated (μm)	Difference |*C* − *O*| (μm)
428 mm	Subregion 1 (Radial distance = 13 mm)	− 0.31	− 0.3	0.01	− 0.34	0.03
	Subregion 2 (Radial distance = 26 mm)	− 20.25	− 20.09	0.16	− 17.63	2.62
	Subregion 3 (Radial distance = 39 mm)	− 31.95	− 30.85	1.1	− 28.94	3.01
	Subregion 4 (Radial distance = 52 mm)	− 37.35	− 36.05	1.3	− 32.67	4.68
448 mm	Subregion 1 (Radial distance = 14 mm)	− 0.23	− 0.22	0.01	− 0.21	0.02
	Subregion 2 (Radial distance = 28 mm)	− 14.42	− 13.78	0.64	− 12.9	1.52
	Subregion 3 (Radial distance = 42 mm)	− 24.31	− 3.23	1.08	− 21.83	2.48
	Subregion 4 (Radial distance = 56 mm)	− 27.97	− 26.22	1.75	− 24.76	3.21

## Supplementary Materials

The Supplementary Materials are available online at http://www.mdpi.com/1424-8220/19/3/744/s1.
