# Supplementary material for: A Knowledge-Driven Approach for 3D High Temporal-Spatial Measurement of an Arbitrary Contouring Error of CNC Machine Tools Using Monocular Vision
Source: Sensors (Basel). 2019 Feb 12;19(3):744. doi: 10.3390/s19030744 (PMC6387178; doi:10.3390/s19030744)
Supplement: Supplementary file 1 [file sensors-19-00744-s001.ppt]

## Slide 1
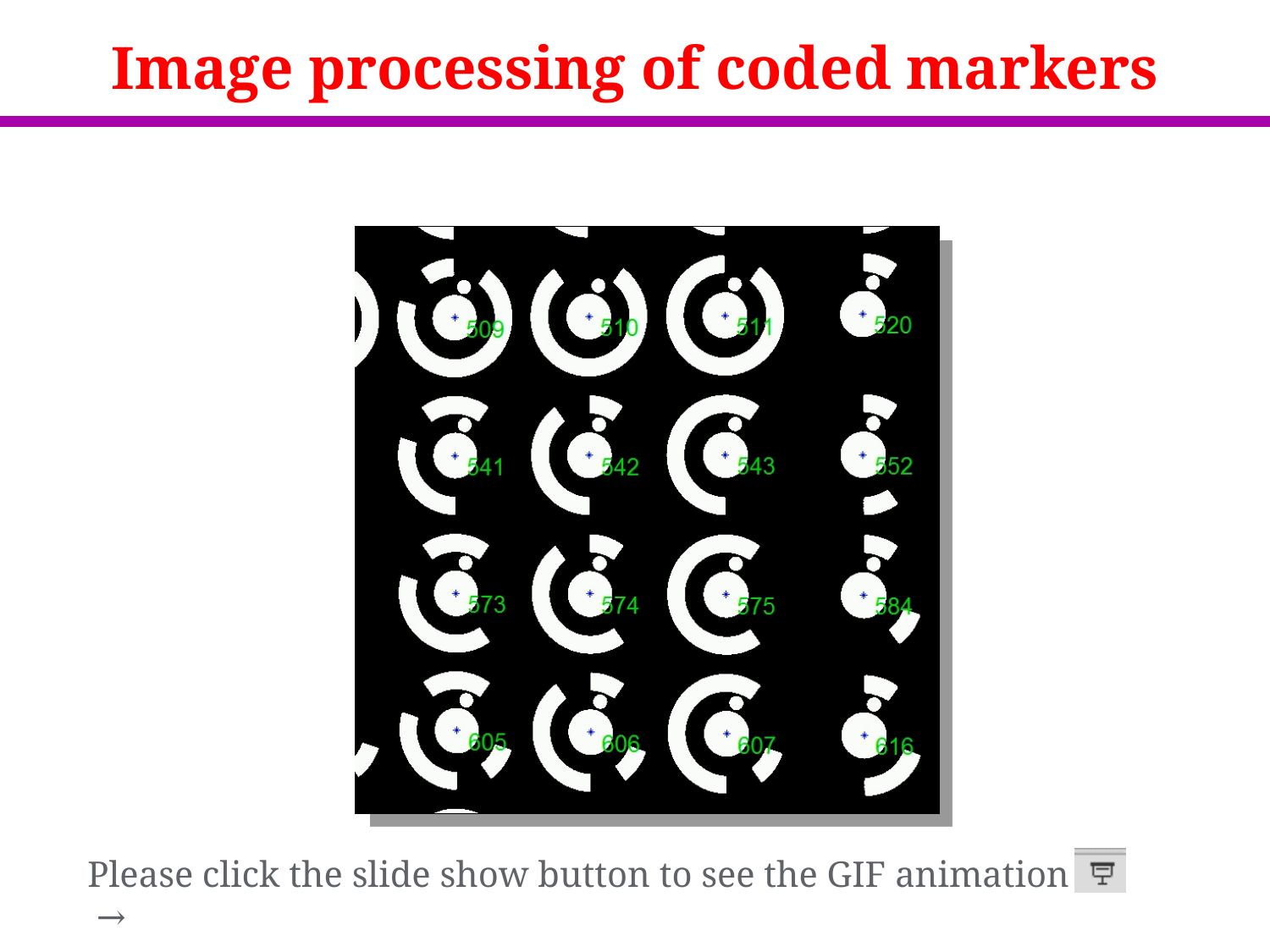

Image processing of coded markers
Please click the slide show button to see the GIF animation →
